# Carbohydrate antigen microarray analysis of serum IgG and IgM antibodies before and after adult porcine islet xenotransplantation in cynomolgus macaques

**DOI:** 10.1371/journal.pone.0253029

**Published:** 2021-06-17

**Authors:** Yoshihide Nanno, Eric Sterner, Jeffrey C. Gildersleeve, Bernhard J. Hering, Christopher Burlak

**Affiliations:** 1 Department of Surgery, Schulze Diabetes Institute, University of Minnesota, Minneapolis, Minnesota, United States of America; 2 Chemical Biology Laboratory, Center for Cancer Research, National Cancer Institute, Frederick, Maryland, United States of America; Imperial College Healthcare NHS Trust, UNITED KINGDOM

## Abstract

Understanding the anti-carbohydrate antibody response toward epitopes expressed on porcine cells, tissues, and organs is critical to advancing xenotransplantation toward clinical application. In this study, we determined IgM and IgG antibody specificities and relative concentrations in five cynomolgus monkeys at baseline and at intervals following intraportal xenotransplantation of adult porcine islets. This study utilized a carbohydrate antigen microarray that comprised more than 400 glycoconjugates, including historically reported α-Gal and non-α-Gal carbohydrate antigens with various modifications. The elicited anti-carbohydrate antibody responses were predominantly IgM compared to IgG in 4 out of 5 monkeys. Patterns of elicited antibody responses greater than 1.5 difference (log2 base units; 2.8-fold on a linear scale) from pre-serum to post-serum sampling specific for carbohydrate antigens were heterogeneous and recipient-specific. Increases in the elicited antibody response to α-Gal, Sd^a^, GM2 antigens, or Lexis X antigen were found in individual monkeys. The novel carbohydrate structures Galβ1-4GlcNAcβ1-3Galβ1 and *N*-linked glycans with Manα1-6(GlcNAcβ1-2Manα1–3)Manβ1-4GlcNAcβ structure were common targets of elicited IgM antibodies. These results provide important insights into the carbohydrate epitopes that elicit antibodies following pig-to-monkey islet xenotransplantation and reveal possible targets for gene editing.

## Introduction

Islet transplantation is the most effective treatment option for type 1 diabetes patients with severe glycemic instability. Excellent short-term results in islet allotransplantation have been reported [1], however, the shortage of human donors and poor long-term outcomes demand an alternative [2]. Pigs are a potential supply of donor organs and cells for xenotransplantation into humans, but preclinical data of efficacy and safety in monkey models is incomplete. Antibody recognizing cell surface carbohydrates and proteins of xenogeneic grafts activate the classical complement pathway, which leads to acute and chronic rejection of the graft [3–9]. The predominant carbohydrate xenoantigen is the galactose-α-1,3-galactose (Galα1-3Gal, α-Gal) epitope, which is synthesized by the α1,3-galactosyltransferase gene (*GGTA1*) and a target of hyperacute rejection (HAR) [10–12]. In addition to α-Gal, *N*-glycolylneuraminic acid (Neu5Gc) and the Sd^a^ glycan are also known xenoantigens that contribute to early antibody-mediated immune injury [13–15].

Recent progress in genome editing has paved the way for the creation of pigs whose cells have reduced levels of the known xenoreactive antigens, which prolonged xenograft survival [16, 17]. Nevertheless as-yet-discovered xenoantigens still prevented graft acceptance [15]. Identifying additional novel xenoantigens will play an important role in developing strategies for establishing long-term graft function. We have recently performed a detailed analysis of natural IgM and IgG antibody specificity and concentration from naïve cynomolgus macaques utilizing a novel carbohydrate microarray (glycan microarray), in which more than 400 glycoconjugates and carbohydrates are attached to solid supports [18]. Use of high-resolution carbohydrate microarray has the potential to elucidate the specific carbohydrate target of rejection by directly comparing naïve and posttransplant sera from individual monkeys. The current study revealed that (i) the elicited anti-carbohydrate antibody responses were predominantly IgM compared to IgG; (ii) patterns of antibody responses were heterogeneous and individually specific; (iii) the novel carbohydrate structures Galβ1-4GlcNAcβ1-3Galβ1 and Manα1-6(GlcNAcβ1-2Manα1–3)Manβ1-4GlcNAcβ were common targets of elicited IgM. This study reveals potential targets of genetic engineering that could give rise to donor animals that have lower antibody binding or as novel targets of screening tools to select low antibody reactivity recipients.

## Materials and methods

### Animals

We have previously performed a detailed analysis for natural IgM and IgG antibody levels of naïve cynomolgus macaques (*Macaca fascicularis*) of Mauritian origin [18]. Five male monkeys with a median age of 5.3 years (range: 4.4–5.8) and a median weight of 5.0 kg (4.4–7.6) with blood type O (*n* = 2), B (*n* = 2), and AB (*n* = 1) were recipients of adult porcine islet xenografts. The housing conditions of the monkeys were described previously [18]. Briefly, They were housed in same-sex pairs or small groups. They had free access to water. Their diet consisted of biscuits (2055 Teklad Global 25% Protein Primate Diet or 7195 Teklad Hi-Fiber Primate Diet; Envigo, Madison, WI) based on body weight, and was enriched daily with fresh fruits, vegetables, grains, beans, nuts, and a multivitamin supplement. Monkeys participated in an environmental enrichment program that included social play, toys, music, and regularly scheduled access to large exercise and swimming areas. All monkeys were also trained to cooperate in medical procedures including blood collection. All animal procedures were approved by the University of Minnesota Institutional Animal Care and Use Committee and conducted in compliance with the Animal Welfare Act and adhere to principles stated in the Guide for Care and Use of Laboratory Animals (Protocol Number: 1404-31481A).

### Pig islet isolation and transplantation

Adult porcine islets were isolated as previously described [19, 20]. Briefly, donor pancreases were retrieved from exsanguinated pigs, dissected, distended intraductally with collagenase and neutral protease, and dissociated using the automated method at 28° to 32°C [21]. Liberated islets were separated from non-islet tissue on continuous density gradients on a COBE 2991 cell separator (Terumo BCT, Lakewood, CO) and cultured free-floating in Medium 199 (Sigma-Aldrich, St. Louis, MO) for 7 days before being infused intraportally through an indwelling catheter at a dose of 25,000 islet equivalents per kg into streptozotocin (100 mg/kg i.v.)-diabetic monkeys [22]. One monkey (13GP08, O) received islets from *GGTA1*-KO porcine donors, and the others received islets from wild-type porcine donors. Immunosuppression was administered to all recipient NHPs as described previously with modification (manuscript in preparation) [22]. Recipient monkey monitoring included daily clinical assessments by study staff, regular evaluations by veterinary staff, and weekly hematology and chemistry laboratory studies. Blood samples were taken from a totally implantable venous access port as previously described [18]. Posttransplant serum samples were collected at the time of clinical rejection (posttransplant day 142, 46, 42, 28, and 35 for monkey 13GP08, 12JP01, 13CP03, 13CP10, and 13GP10, respectively). Collected blood samples were allowed to clot for 30 min in sterile additive-free collection tubes at room temperature, centrifuged with 2000 *g* for 10 minutes, stored at −80°C, and shipped to Chemical Biology Laboratory, Center for Cancer Research, National Cancer Institute (Frederick, MD) for further analysis. Monkeys were euthanized under anesthesia with Beuthanasia-D (390mg pentobarbital plus 50mg phenytoin; Schering-Plough Animal Health, Kenilworth, NJ), followed by a necropsy to confirm the rejection by histology.

### Carbohydrate microarray binding assay

All sample analyses were performed at the Chemical Biology Laboratory, Center for Cancer Research, National Cancer Institute (Frederick, MD). Carbohydrate microarray fabrication was performed as previously described [23, 24], and variability of the microarray assay has been evaluated previously [24–26]. Briefly, serum samples were profiled on a carbohydrate array (version A411) containing 408 carbohydrates and glycopeptides which were conjugated to albumin to produce neoglycoproteins [27]. The number after the abbreviation indicates the average number of carbohydrates per molecule of albumin. Each monkey serum sample was assayed in two separate wells for IgG and two separate wells for IgM. All serum samples were diluted 1:50 in 3% BSA (w/v, Sigma-Aldrich), 1% HSA (Sigma-Aldrich) in PBST [PBS with 0.05% (v/v) Tween 20 (Sigma-Aldrich)], and 100 μL of each sample was added to arrays. Bound monkey antibodies were detected by incubating with tetramethylrhodamine isothiocyanate (TRITC) goat anti-monkey IgG (Brookwood Biomedical, Jemison, AL) or TRITC anti-monkey IgM (Brookwood Biomedical) at a dilution of 1:250 in 1% BSA, 3% HSA in PBST at 37°C with gentle shaking at 100 rpm (approximately 1 *g*) for 2 hours. All clinical/demographic information was blinded during the profiling of serum samples.

Slides were scanned at 5 μm resolution with an InnoScan 1100 AL fluorescence scanner (Innopsys, Carbonne, France), and image analysis was carried out with Genepix Pro 6.0 analysis software (Molecular Devices Corporation, Union City, CA) as previously described [23, 24]. To minimize the impact of noise on our comparisons, spots with intensity lower than 150 (1/2 the typical background signal when analyzing IgM and IgG at 1:50) were rounded to 150. The average of duplicate spots was calculated to obtain a normalized value to the reference samples and shown in a log-transformed (base 2) form, which enables a direct comparison of values from one experiment to another. Full microarray data can be found in the S1 Table. The normalized anti-carbohydrate antibody signal intensities were summarized as median and standard deviation.

## Results

### Anti-carbohydrate antibody responses in posttransplant sera

**Fig 1:**
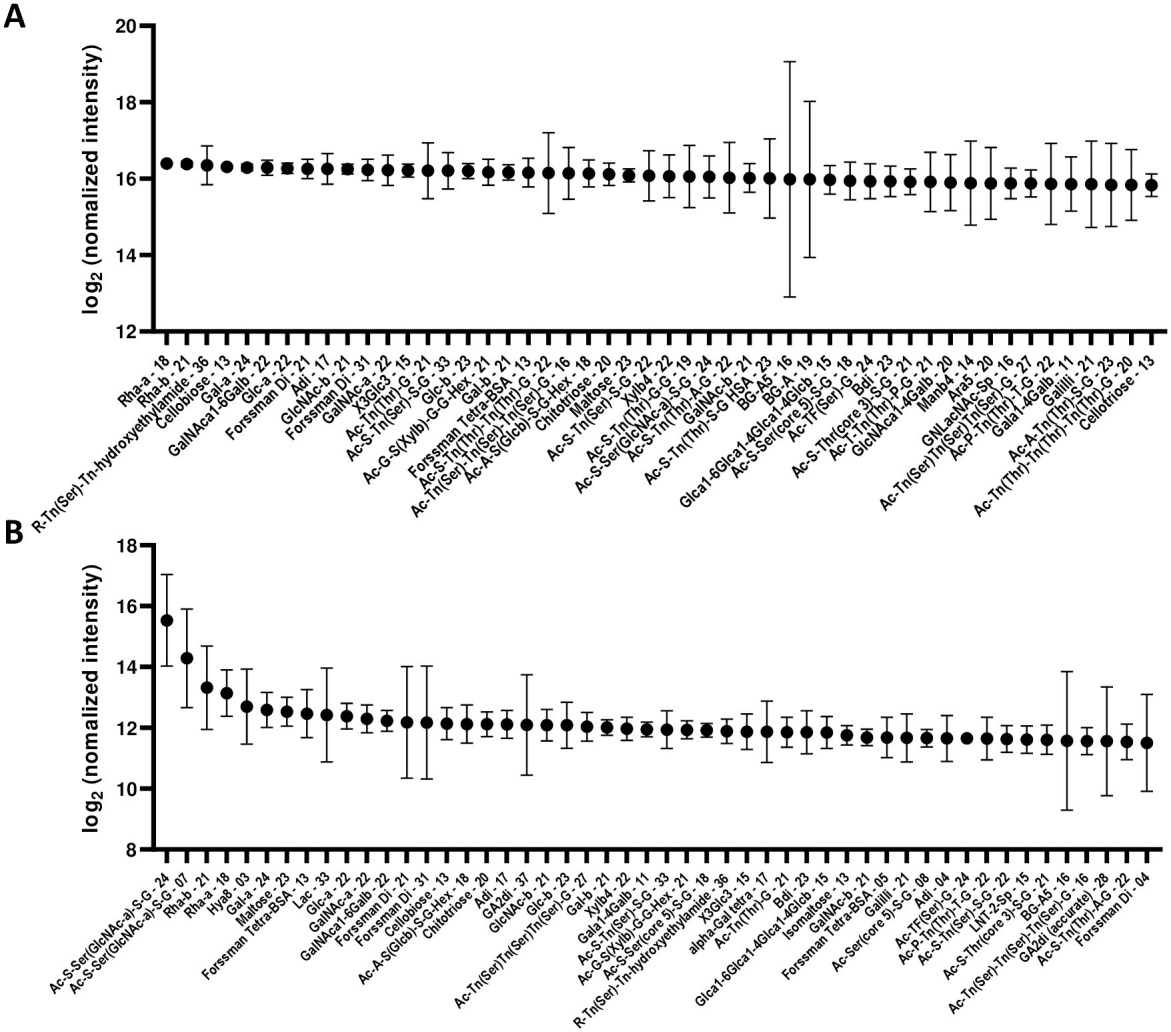
The distribution (log-transformed base 2) of 50 highest serum IgM (A) and IgG (B) elicited antibody signals in five monkeys. The number after the abbreviation indicates the average number of carbohydrates per molecule of albumin. The circle represents the median of the five monkeys, and the bars represent the standard deviation for the group.

The median of IgM and IgG signals were 12.50 (7.23–16.40) and 9.23 (7.23–15.53), respectively. The distribution of 50 highest antibody signals in the posttransplant sera for each array component (*n* = 408) is shown in Fig 1A (IgM) and 1B (IgG). The details of signal intensities are shown in S1 Table. The distribution of anti-carbohydrate antibody signal intensity in the sera was mostly similar to that of naïve sera in the previous report [18], but blood group A antigens (BG-A5-16, and BG-A-19) were listed in the posttransplant IgM and IgG repertoires with a wide distribution (Fig 1).

### Comparison of antibody responses between naïve and posttransplant sera

**Fig 2:**
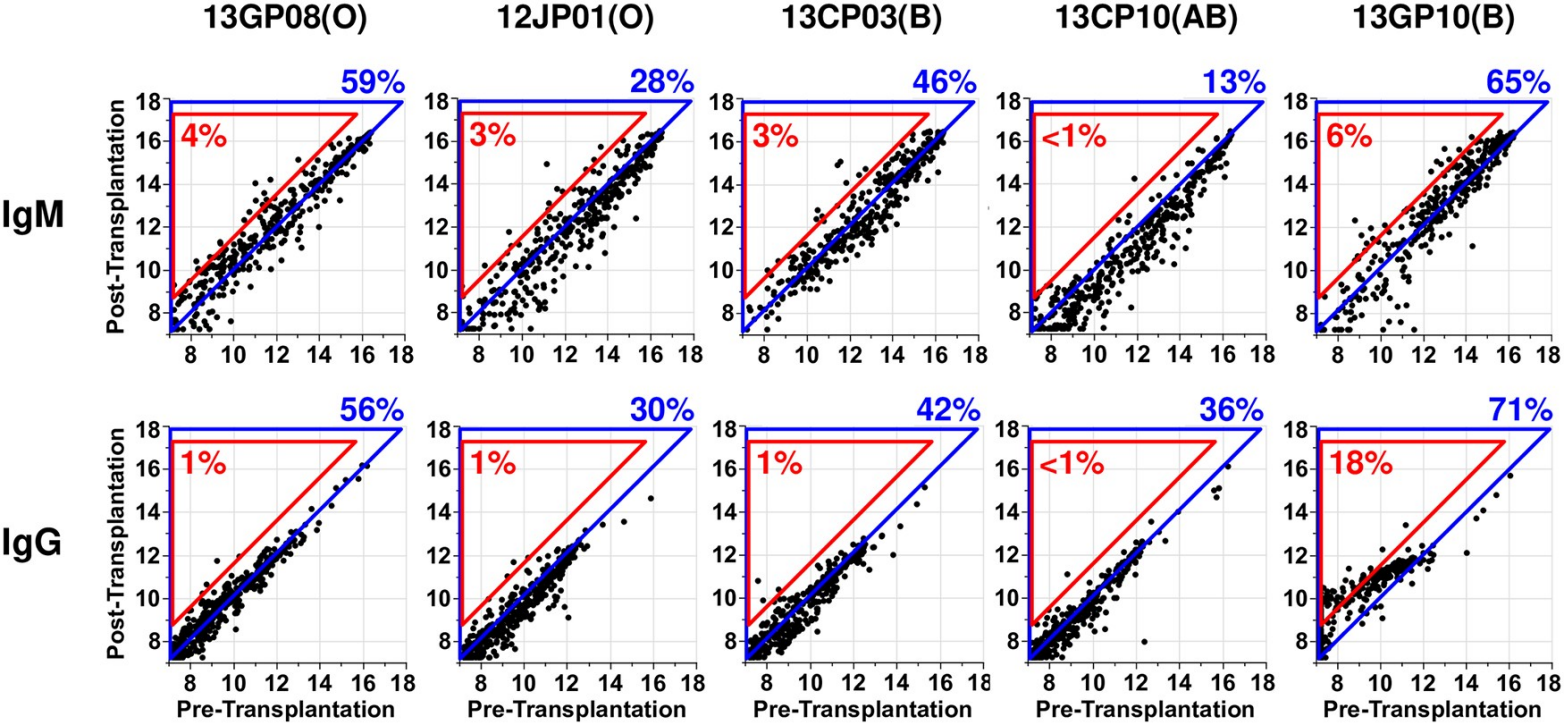
Comparison of antibody signals in each monkey. Signals for each carbohydrate in posttransplant serum were plotted against pretransplant naïve serum. Signals are on the log 2 scale. The plots in a blue triangle represent carbohydrate antigens that are increased in the posttransplant serum. The plots in a red triangle represent carbohydrate antigens that are significantly increased (the changes of normalized signal intensity ≥1.5) in the posttransplant serum. The blood type of a monkey is noted in brackets.

**Fig 3:**
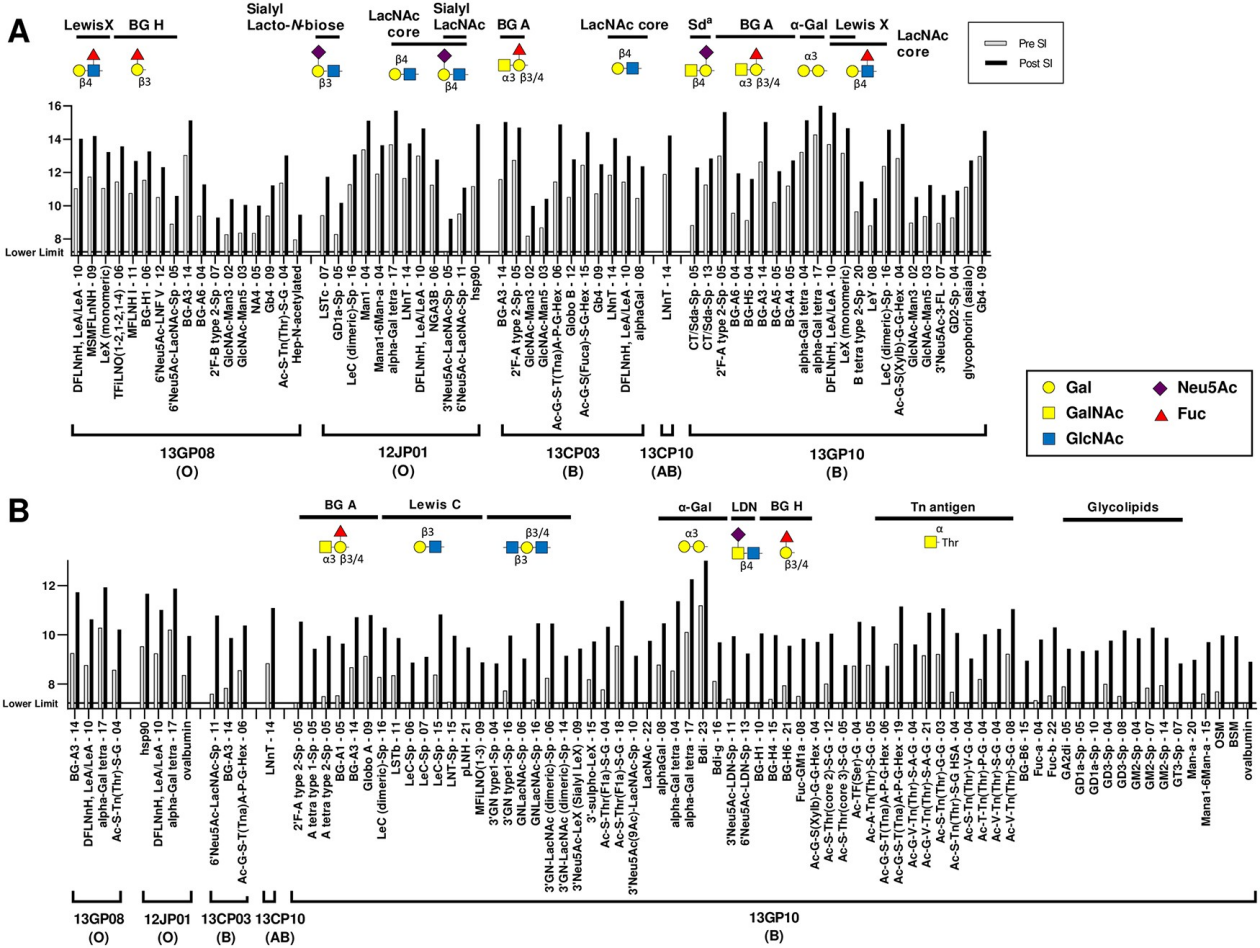
The list of carbohydrates that were significantly elicited in the posttransplant sera (the changes of normalized signal intensity ≥1.5; naïve sera, gray; posttransplant sera, black). (A) IgM repertoire. (B) IgG repertoire. The number after the abbreviation indicates the average number of carbohydrates per molecule of albumin. The blood type of a monkey is noted in brackets.

**Fig 4:**
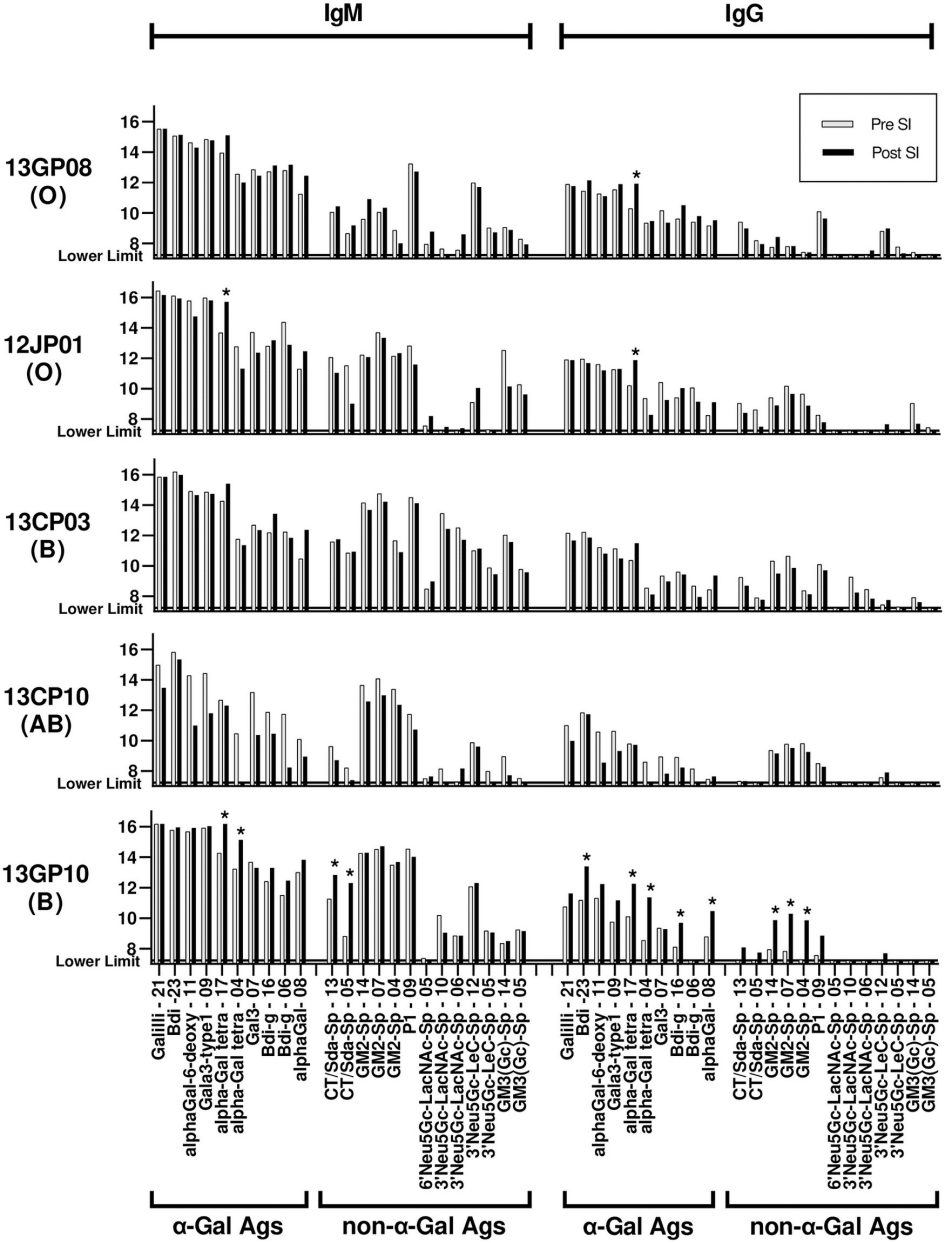
The list of antibody signals against historically reported α-Gal and non-α-Gal carbohydrate antigens in IgM and IgG repertoires. The α-Gal antigens include carbohydrate structures with Galα1-3Gal epitope. The non-α-Gal antigens include carbohydrate structures with Sd^a^ and Neu5Gc epitopes. The number after the abbreviation indicates the average number of carbohydrates per molecule of albumin. An antibody signal that is significantly increased (the changes of normalized signal intensity ≥1.5) in the posttransplant serum is marked with an asterisk.

The comparison of IgM and IgG signal intensities between pretransplant naïve sera and posttransplant sera are shown in Fig 2. The changes of normalized signal intensity ≥1.5 (log base 2 units; 2.8-fold on a linear scale) from pretransplant naïve serum IgM and IgG were considered significant. The threshold was determined by the previous analysis showing that the incidence of 1.5 signal intensity difference between the two time points in the one-tailed test was approximately 1% for both IgG and IgM antibodies [25]. The carbohydrates with a significant difference were grouped by epitope types and listed in Fig 3. The analysis of IgM and IgG signal intensities for previously reported α-Gal and non-α-Gal carbohydrate antigens were summarized in Fig 4.

#### Monkey 13GP08 (blood group O)

Anti-carbohydrate antibodies exhibited a signal intensity rise against approximately 60% of all carbohydrates in both IgG and IgM (Fig 2). Antibody signal intensities against 17 carbohydrates were significantly elevated (cutoff of 1.5), which included carbohydrates with Lewis X and blood group H type-1 structures in IgM, whereas a specific trend was not observed in the IgG repertoire (Fig 3). The antibody response toward an α-Gal epitope (alpha-Gal tetra-17) was elevated in IgG, but the antibody responses toward other related α-Gal epitopes were not elevated in IgM and IgG repertoires (Fig 4).

#### Monkey 12JP01 (blood group O)

The most unique anti-carbohydrate antibody reactivity detected in the sera was a significant response against Sialyl Lacto-*N*-biose and Sialyl *N*-acetyllactosamine (LacNAc) structures (Neu5Acα2-6Galβ1-3/4GlcNAc) in IgM (Fig 3). This monkey exhibited an increase in signal intensity towards approximately 30% of carbohydrates in both IgM and IgG repertoires (Fig 2). Elicited antibody from 12JP01, as in 13GP08, recognized the alpha-Gal tetra-17 carbohydrate in addition to previously unrecognized non-α-Gal carbohydrate structures.

#### Monkey 13CP03 (blood group B)

Antibody from 13CP03 revealed a vigorous anti-blood group A antibody response and anti-LacNAc core structure response (Fig 3). The antibody signal intensities against blood group A antigens were significant (15.04–15.86) in the IgM repertoire. No specific trend was not observed in the IgG repertoire.

#### Monkey 13CP10 (blood group AB)

13CP10 received islets from the same donor as 12JP01. In contrast to the other monkeys, a majority of carbohydrate signal intensities were decreased in both IgM and IgG repertoires (Fig 2). A significant signal increase against LNnT-14 (Lacto-*N*-neotetraose, Galβ1-4GlcNAcβ1-3Galβ1-BSA) was detected in both IgM and IgG repertoires (Fig 3). Of note, the signal against LNnT-14 was also elicited in 12JP01 and 13CP03. However, LNnT-04, which possesses the same epitope but in lower density, was not recognized by antibody in monkeys suggesting that antigen density is important in recognition.

#### Monkey 13GP10 (blood group B)

The IgM binding significantly increased against 65% of all carbohydrates and IgG increased 71% (Fig 2). The elicited antibody response included Sd^a^ antigen, blood group A antigen, α-Gal antigen, and Lewis X antigen. The GM2 antigen that shares the Sd^a^ epitope [Neu5Acα2-3(GalNAcβ1–4)Gal] showed a signal increase in the IgG repertoire. The GlcNAcβ1-3Galβ1-3/4GlcNAc structure, LDN, and Tn antigen carbohydrate structures also showed increases in the IgG response.

### Carbohydrate antigens elicited in common among sera of five monkeys

**Fig 5:**
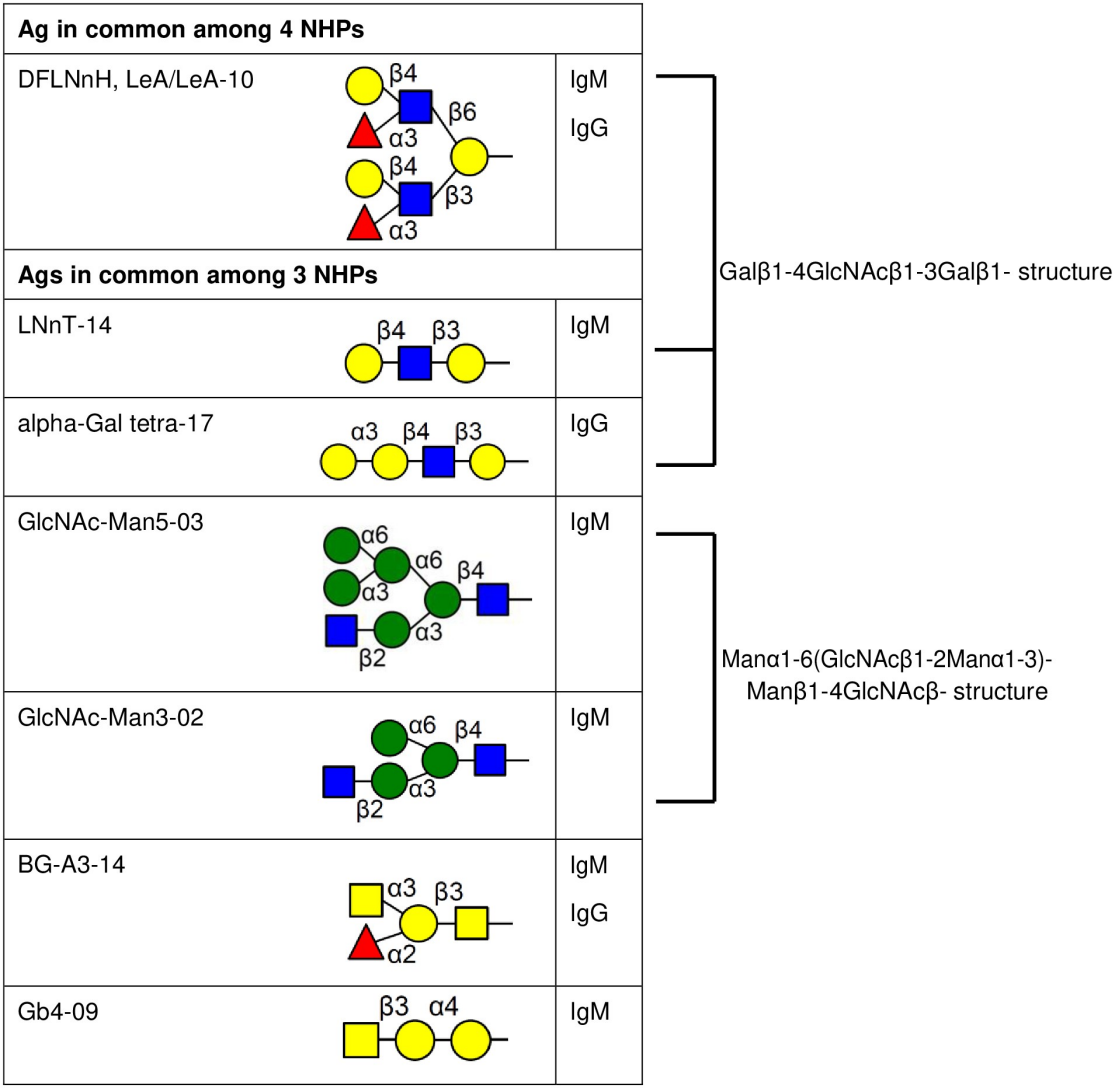
Carbohydrate antigens elicited in common among sensitized monkey sera.

Fig 5 summarizes the carbohydrate targets of anti-carbohydrate antibodies elicited among five monkeys. The LNnT epitope (Galβ1-4GlcNAcβ1-3Galβ1), shared among DFLNnH LeA/LeA, LNnT, and α-Gal tetra epitopes, elicited antibody responses in all monkeys. Two *N*-linked glycans in the figure shared the GlcNAc-Man3 structure [Manα1-6(GlcNAcβ1-2Manα1–3)Manβ1-4GlcNAcβ]. The signal intensities against the LNnT epitope were stronger than those against the GlcNAc-Man3 structures (Fig 3). No elicited anti-Neu5Gc response was detected in monkeys (Fig 4).

## Discussion

Carbohydrate antigens on the cell surface have been shown to be major xenoantigens [28, 29]. Genetic deletion of the enzymes that mediate the creation of those structures has reduced antibody binding, overcoming HAR [6]. Yet flow cytometric crossmatch analysis suggests that additional antibody targets continue to contribute to xenograft rejection [15]. The current carbohydrate antigen microarray study lead us to conclude that the antibody responses against carbohydrate epitopes after pig-to-monkey xenotransplantation: (i) are predominantly IgM compared to IgG in 4 out of 5 monkeys; (ii) are unique and specific to individual monkeys; only 1 out of 5 monkeys (13GP10) transplanted wild-type adult porcine islet had a statistically significant response against α-Gal and Sd^a^ antigens; (iii) yet the novel carbohydrate antigen structures LNnT (Galβ1-4GlcNAcβ1-3Galβ1) and GlcNAc-Man3 [Manα1-6(GlcNAcβ1-2Manα1–3)Manβ1-4GlcNAcβ] were targets of elicited IgM antibodies in 5 out of 5 and 3 out of 5 monkeys, respectively.

Blixt *et al*. performed a detailed characterization of the elicited human antibodies after transplantation of porcine fetal islet-like cell clusters using a carbohydrate array system [29]. Our current array study further assessed sera after adult porcine islet transplantation and revealed novel antibody responses including those against LNnT and GlcNAc-Man3 structures, which were not found in the previous studies. It is notable that the presence of high-mannose type *N*-glycans with GlcNAc-Man3 structure has recently been confirmed on adult porcine islet [30]. In the study, we performed a mass spectrometric analysis of glycan structures on adult porcine islet and confirmed that NeuAc-Gal-GlcNAc-Man5 structure was exclusively detected from porcine islets but not from human islets by mass spectrometry [30]. Our current study also suggested that the elicited antibody response to LNnT is density specific; only directed toward LNnT with higher density (LNnT-14 and DFLNnH, LeA/LeA-10) but not with lower density (LNnT-04). As antibodies achieve tight binding through formation of multivalent complexes, the density and the spacing between glycans affect the ability to form multivalent interactions [26]. Our previous studies have suggested that genetic modification of *GGTA1* and *CMAH* could interfere with normal carbohydrate synthesis and cause accumulation of incomplete carbohydrate components including LNnT and high mannose incomplete *N*-glycans [31–33], presumably with a greater response to these structures in genetically modified donors. Muthana and Gildersleeve have measured the presence of anti-LNnT (Galβ1-4GlcNAcβ1-3Galβ1) and anti-GlcNAc-Man3 [Manα1-6(GlcNAcβ1-2Manα1–3)Manβ1-4GlcNAcβ] antibodies in human sera using the same glycan array used in our study [23]. Humans naturally have antibodies against the LNnT structure called cold agglutinins and the antibody has been thought to be irrelevant at body temperature and does not influence the outcome of the xenotransplantation [23, 24], however, the impact of these antibodies at the time of infusion or transplant should be evaluated in the future studies. The successful generation of an LNnT deficient mouse was reported by deletion of β1,3-N-acetylglucosaminyltransferase 5 gene (*B3gnt5*) [34]. Deletion of this gene may reduce the antigenicity of the xenograft.

Although about 80% of human anti-porcine natural antibody is directed toward the α-Gal antigen that directly contributes to HAR [35], previous reports have shown that adult islet cells express negligible amount of α-Gal antigen [36] and a long-term reversal of diabetes is achieved after islet xenotransplantation in wild type pig-to-NHP models [37]. However, in the current study, 3 out of 5 monkeys had elicited antibody responses against the same single α-Gal antigen (alpha-Gal tetra, Galα1-3Galβ1-4GlcNAcβ1-3Galβ1). This is likely a part of the antibody responses against Galβ1-4GlcNAcβ1-3Galβ1 core since the same response was also observed in the recipient of islets from *GGTA1*-KO pig and antibody response against other α-Gal epitopes were not observed in these monkeys (Fig 4). Sd^a^ antigen was identified as a non-α-Gal antigen by Byrne *et al*. [38] and the elimination of the Sd^a^ epitope significantly reduced the level of human antibody specific for pig cells [15]. Our study showed basal levels of specific antibody binding to Sd^a^ and related GM2 antigens, however, a significant increase in the elicited antibody response was observed in only one monkey (13GP10). Elicited antibody responses were also observed against blood group A antigens though not against B antigens (Figs 1 and 3). It is previously reported that there are only A or O blood types in pigs [39]. Although we did not perform blood type testing of donor pigs at the time these transplants occurred, we assume that the sensitization could occur from donor pig antigens. Cynomolgus macaques express the Neu5Gc epitope, which is analogous to pigs but naturally absent in humans. As expected, epitopes with terminal Neu5Gc did not show a significant antibody response (Fig 4). Statistically significant elicited antibody responses were predominantly IgM except 13GP10 (Fig 3). Signal increases in IgG as compared to IgM for the same glycan structure were observed, such as LNnT (*e*.*g*. 13CP10), suggesting that there is evidence of class switching yet further analysis is required to draw significant conclusions.

Recently, alternative and novel immunological pathways have been discovered and utilized to improve islet transplantation outcomes [40]. Multidisciplinary approaches using these new immunoregulatory technologies in combination with optimized recipients with low reactivity towards xenoantigens will make it possible to progress clinical islet xenotransplantation to reality.

Several limitations of our study should be noted. First, our array did not cover all carbohydrate antigens that may present on the graft. Our observations and conclusions may lack insights into these antigens/antibodies. Second, the study analyzed only a limited number of monkeys that were transplanted with wild-type or *GGTA1*-KO porcine islet grafts. Larger studies with possibly multiple-gene KO porcine donors would shed light on the specific antibody response to genetically modified pigs that are proposed to be used in clinical trials. Third, our array is a model that mimics natural presentation but not identical to the natural system. Positive antibody binding does not mean that it will always occur in the physiological setting.

In conclusion, our study highlights the important implications for a comprehensive understanding of pre-existing and elicited antibody responses to xenografts. A heterogeneous and individually specific elicited antibody response suggests that the careful selection of recipients for low reactive antibody could positively affect outcomes in xenograft studies. Additional characterization of the identified novel carbohydrate antigens LNnT (Galβ1-4GlcNAcβ1-3Galβ1) and GlcNAc-Man3 [Manα1-6(GlcNAcβ1-2Manα1–3)Manβ1-4GlcNAcβ] will be required to validate their contribution to xenograft rejection.

## Supporting information

S1 Table List of serum IgM and IgG natural antibody signals from five cynomolgas macaques to 408 array components. (DOCX)
