# Supplementary material for: Carbohydrate antigen microarray analysis of serum IgG and IgM antibodies before and after adult porcine islet xenotransplantation in cynomolgus macaques
Source: PLoS One. 2021 Jun 17;16(6):e0253029. doi: 10.1371/journal.pone.0253029 (PMC8211184; doi:10.1371/journal.pone.0253029)
Supplement: S1 Table — (DOCX) [file pone.0253029.s001.docx]

S1 Table. List of serum IgM and IgG natural antibody signals from five cynomolgas macaques to 408 array components

| **a411 print #** |  |  | IgM signal | | | | |  | IgG signal | | | | |
| --- | --- | --- | --- | --- | --- | --- | --- | --- | --- | --- | --- | --- | --- |
|  | **Abbreviation** | **Description** | 13GP08 | 12JP01 | 13CP03 | 13CP10 | 13GP10 |  | 13GP08 | 12JP01 | 13CP03 | 13CP10 | 13GP10 |
| 1 | Cy3 | Cy3-BSA (20mg/mL + BSA, 125mg/mL total) | 17.5 | 17.5 | 17.6 | 17.6 | 17.5 |  | 17.4 | 17.5 | 17.3 | 17.6 | 17.5 |
| 2 | BSA | Bovine serum albumin | 9.3 | 8.9 | 9.5 | 8.9 | 8.9 |  | 9.4 | 9.2 | 9.6 | 9.7 | 8.2 |
| 3 | Maltose - 23 | Glca1-4Glcb-BSA | 16.1 | 16.4 | 16.0 | 16.1 | 16.3 |  | 12.5 | 12.6 | 12.0 | 13.1 | 11.9 |
| 4 | Glc-b - 23 | Glc-b - BSA | 16.1 | 16.3 | 15.9 | 16.4 | 16.2 |  | 12.8 | 12.1 | 11.7 | 12.4 | 10.8 |
| 5 | GalNAc-a - 22 | GalNAc-a - BSA | 16.3 | 16.2 | 16.2 | 15.4 | 16.3 |  | 13.1 | 12.3 | 12.3 | 12.0 | 11.9 |
| 6 | Glc-a - 22 | Glc-a - BSA | 16.2 | 16.4 | 16.3 | 16.0 | 16.3 |  | 12.4 | 12.4 | 11.7 | 12.5 | 11.6 |
| 7 | Man-a - 20 | Man-a - BSA | 14.9 | 15.3 | 15.5 | 10.5 | 15.2 |  | 10.9 | 11.1 | 10.8 | 8.2 | 9.0 |
| 8 | GlcNAc-b -21 | GlcNAc-b - BSA | 16.3 | 16.4 | 16.3 | 16.0 | 16.2 |  | 12.7 | 12.4 | 11.7 | 12.1 | 11.4 |
| 9 | GalNAc-b - 21 | GalNAc-b - BSA | 15.5 | 16.1 | 16.0 | 15.3 | 16.1 |  | 11.6 | 12.2 | 11.8 | 11.7 | 11.4 |
| 10 | Fuc-b - 22 | Fuc-b - BSA | 14.8 | 15.5 | 16.0 | 13.0 | 16.2 |  | 10.5 | 10.7 | 11.8 | 9.5 | 10.3 |
| 11 | Fuc-a - 22 | Fuc-a - BSA | 15.0 | 16.1 | 15.8 | 14.6 | 15.8 |  | 11.0 | 11.9 | 11.5 | 11.2 | 10.9 |
| 12 | Rha-a - 18 | Rha-a – BSA | 16.4 | 16.4 | 16.4 | 16.4 | 16.3 |  | 13.1 | 13.1 | 14.3 | 14.0 | 12.4 |
| 13 | Rha-b - 21 | Rha-b - BSA | 16.4 | 16.4 | 16.4 | 16.2 | 16.4 |  | 15.5 | 12.7 | 13.3 | 13.5 | 11.8 |
| 14 | Gal-a- 24 | Gal-a - BSA | 16.1 | 16.4 | 16.3 | 16.2 | 16.3 |  | 13.4 | 12.4 | 12.9 | 12.6 | 11.9 |
| 15 | Gal-b- 21 | Gal-b - BSA | 16.2 | 16.1 | 16.4 | 15.9 | 16.3 |  | 12.0 | 11.9 | 12.2 | 12.0 | 11.5 |
| 16 | Ac-Tn(Thr)-G - 21 | Ac(GalNAca)Thr-Gly-Hex-BSA | 16.4 | 15.2 | 16.2 | 14.8 | 16.3 |  | 12.7 | 11.4 | 11.9 | 11.6 | 11.9 |
| 17 | GalNAca1-6Galb - 22 | GalNAca1-6Galb-BSA | 16.4 | 16.2 | 16.3 | 15.9 | 16.4 |  | 12.6 | 12.2 | 12.3 | 12.2 | 11.7 |
| 18 | Adi - 17 | GalNAca1-3Galb-BSA | 16.3 | 16.2 | 16.4 | 15.4 | 16.3 |  | 12.9 | 12.1 | 12.9 | 12.0 | 12.1 |
| 19 | GlcNAca1-4Galb - 20 | GlcNAca1-4Galb-BSA | 14.9 | 16.2 | 15.9 | 14.7 | 16.2 |  | 12.5 | 12.2 | 11.3 | 11.1 | 11.3 |
| 20 | Bdi -23 | Gala1-3Gal– BSA | 15.1 | 15.9 | 16.0 | 15.3 | 15.9 |  | 12.1 | 11.7 | 11.9 | 11.7 | 13.4 |
| 21 | LacNAc - 22 | Galb1-4GlcNAc – BSA | 12.0 | 12.5 | 13.7 | 10.9 | 14.8 |  | 9.0 | 9.0 | 9.6 | 8.4 | 9.8 |
| 22 | GA1di -11 | Galb1-3GalNAcb – HSA | 14.4 | 13.8 | 15.1 | 13.6 | 14.3 |  | 10.9 | 10.2 | 10.8 | 10.8 | 9.9 |
| 23 | Lac - 33 | Galb1-4Glcb – BSA | 15.2 | 15.5 | 16.3 | 14.4 | 16.3 |  | 11.2 | 11.2 | 12.4 | 14.7 | 13.7 |
| 24 | Ac-S-Ser(GlcNAc-a)-S-G - 24 | Ac-Ser-(GlcNAcα)Ser-Ser-Gly-Hex-BSA | 15.9 | 16.3 | 16.2 | 14.9 | 16.0 |  | 15.5 | 14.6 | 12.3 | 16.1 | 15.7 |
| 25 | Galb1-6Man-a - 13 | Galb1-6Man-a - BSA | 15.1 | 15.5 | 16.4 | 14.9 | 16.0 |  | 10.9 | 11.3 | 11.9 | 11.3 | 10.9 |
| 26 | Mana1-6Man-a - 15 | Mana1-6Man-a - BSA | 13.4 | 15.4 | 15.7 | 13.4 | 14.7 |  | 13.5 | 11.3 | 11.8 | 10.1 | 9.7 |
| 27 | Ac-S-Thr(F1a)-S-G - 04 | Ac-Ser-(Galb1-4GlcNAcb1-6GalNAca)Thr-Ser-Gly-Hex-BSA | 14.3 | 11.3 | 14.7 | 12.0 | 15.2 |  | 10.8 | 7.7 | 10.8 | 9.6 | 10.3 |
| 28 | Cellobiose -13 | Glcb1-4Glcb-BSA | 16.3 | 16.4 | 16.3 | 16.3 | 16.3 |  | 12.1 | 12.2 | 12.1 | 12.2 | 11.0 |
| 29 | HSA | Human serum albumin (isolated from serum) | 9.4 | 8.2 | 9.0 | 7.7 | 7.2 |  | 7.2 | 7.2 | 7.2 | 7.2 | 7.2 |
| 30 | Isomaltose - 13 | Glca1-6Glcb-BSA | 15.4 | 16.3 | 16.0 | 15.7 | 15.6 |  | 11.2 | 12.1 | 11.6 | 11.9 | 11.8 |
| 31 | Gala1-4Galb - 11 | Gala1-4Galb-CETE-BSA | 15.2 | 15.9 | 16.4 | 14.7 | 16.2 |  | 12.1 | 11.9 | 12.1 | 11.6 | 11.7 |
| 32 | ManT -26 | Mana1-6[Mana1-3]Manb -BSA | 12.0 | 14.0 | 12.8 | 11.6 | 12.7 |  | 9.2 | 9.7 | 8.8 | 8.8 | 7.9 |
| 33 | 3'Neu5Ac-LacNAc -19 | Neu5Aca2-3Galb1-4GlcNAc – BSA | 7.6 | 11.2 | 11.4 | 9.9 | 9.7 |  | 7.2 | 7.8 | 7.8 | 7.7 | 7.2 |
| 34 | BG-A -19 | GalNAca1-3(Fuca1-2)Galb- -BSA | 16.0 | 14.5 | 16.2 | 11.4 | 16.2 |  | 12.1 | 10.6 | 11.7 | 8.6 | 11.4 |
| 35 | 2'F-A type 2-Sp - 13 | GalNAca1-3[Fuca1-2]Galb1-4[Fuca1-3]GlcNAcb-Sp-BSA | 15.7 | 12.1 | 15.9 | 9.5 | 16.1 |  | 12.0 | 8.7 | 11.1 | 7.7 | 11.3 |
| 36 | BG-H1- 20 | Fuca1-2Galb1-3GlcNAcb1-3Galb1-4Glcb–APD-HSA | 13.7 | 11.8 | 11.8 | 11.4 | 12.3 |  | 9.9 | 8.7 | 8.6 | 9.5 | 7.9 |
| 37 | LeA -18 | Galb1-3[Fuca1-4)GlcNAcb1-3Galb1-4Glcb- BSA | 14.1 | 14.8 | 13.1 | 12.4 | 15.8 |  | 10.2 | 10.8 | 9.0 | 9.1 | 10.4 |
| 38 | LeB - 09 | Fuca1-2Galb1-3(Fuca1-4)GlcNAcb1-3Galb1-4Glcb-BSA | 10.7 | 8.6 | 10.7 | 7.4 | 8.0 |  | 7.5 | 7.2 | 7.4 | 7.2 | 7.2 |
| 39 | LeY -08 | Fuca1-2Galb1-4[Fuca1-3)GlcNAc –HSA | 8.1 | 12.6 | 9.4 | 7.2 | 10.4 |  | 7.8 | 9.3 | 7.2 | 7.2 | 7.2 |
| 40 | LeX (monomeric) | Galb1-4[Fuca1-3)GlcNAc-APD-HSA ; SSEA-1; CD-15 | 13.2 | 13.7 | 12.8 | 11.0 | 14.7 |  | 10.2 | 10.5 | 9.2 | 8.5 | 10.3 |
| 41 | 3'Neu5Ac-LeX (Sialyl LeX) - 09 | Neu5Aca2-3Galb1-4[Fuca1-3)GlcNAc – BSA | 9.8 | 12.2 | 9.5 | 10.7 | 13.8 |  | 8.6 | 9.6 | 7.3 | 9.3 | 9.4 |
| 42 | Gb3- 13 | Gala1-4Galb1-4Glc-HSA [aka: Pk or CD77] | 13.9 | 12.8 | 14.5 | 12.5 | 14.7 |  | 10.9 | 9.5 | 10.3 | 9.3 | 12.1 |
| 43 | Xylb4 - 22 | Xylb1-4Xylb1-4Xylb1-4Xylb1-BSA | 15.6 | 16.1 | 16.3 | 14.9 | 16.2 |  | 12.0 | 12.1 | 11.8 | 12.0 | 11.1 |
| 44 | Ara5 - 20 | Araa1-5Araa1-5Araa1-5Araa1-5Araa1-BSA | 14.1 | 16.3 | 16.3 | 15.0 | 15.9 |  | 10.5 | 12.1 | 10.8 | 10.8 | 10.5 |
| 45 | X3Glc3 - 15 | Xyla1-6Glcb1-4(Xyla1-6)Glcb1-4(Xyla1-6)Glcb1-BSA | 16.0 | 16.3 | 16.3 | 16.0 | 16.2 |  | 11.5 | 12.0 | 12.5 | 11.9 | 10.9 |
| 46 | GM3 - 12 | Neu5Aca2-3Galb1-4Glc-APD-HSA (new, IsoSep, bad?) | 10.1 | 13.1 | 10.2 | 14.5 | 8.7 |  | 8.5 | 9.9 | 8.0 | 11.5 | 7.2 |
| 47 | 6'Neu5Ac-Lac | Neu5Aca2-6Galb1-4Glc-APD-HSA | 9.5 | 13.9 | 11.0 | 13.1 | 8.0 |  | 8.3 | 10.3 | 8.2 | 10.5 | 7.2 |
| 48 | 3'Neu5Ac-LeA -12 | Neu5Aca2-3Galb1-3[Fuca1-4)GlcNAcb1-3Galb1-APD-HSA | 10.0 | 7.5 | 10.1 | 8.2 | 9.7 |  | 7.7 | 7.4 | 7.4 | 7.4 | 7.2 |
| 49 | LeX (dimeric) -07 | Galb1-4[Fuca1-3)GlcNAcb1-3Galb1-4(Fuca1-3)GlcNAcb1-3Galb1-APE-BSA | 13.1 | 13.9 | 12.5 | 11.6 | 14.6 |  | 9.7 | 10.2 | 8.7 | 8.8 | 9.6 |
| 50 | Manb4 - 14 | Manb1-4Manb1-4Manb1-4Manb1-BSA | 14.5 | 16.0 | 15.9 | 13.7 | 16.2 |  | 12.0 | 11.9 | 10.9 | 11.1 | 10.7 |
| 51 | G2M4 - 07 | Manb1-4(Gala1-6)Manb1-4(Gala1-6)Manb1-4Manb1-BSA | 13.7 | 15.6 | 15.2 | 12.7 | 16.0 |  | 10.6 | 11.3 | 9.9 | 9.5 | 10.4 |
| 52 | Glca1-6Glca1-4Glca1-4Glcb - 15 | Glca1-6Glca1-4Glca1-4Glcb-CETE-BSA | 15.7 | 16.4 | 16.0 | 15.4 | 16.0 |  | 11.8 | 12.3 | 12.0 | 11.8 | 10.9 |
| 53 | LNT - 21 | Galb1-3GlcNAcb1-3Galb-BSA | 13.2 | 13.5 | 14.5 | 11.5 | 14.9 |  | 10.3 | 10.2 | 10.3 | 8.4 | 10.2 |
| 54 | LSTa - 10 | Neu5Aca2-3Galb1-3GlcNAcb1-3Galb1-BSA | 10.0 | 11.0 | 11.6 | 9.6 | 11.2 |  | 7.5 | 8.0 | 8.2 | 7.7 | 7.2 |
| 55 | LSTb - 11 | Galb1-3(Neu5Aca2-6)GlcNAcb1-3Galb1-BSA | 12.3 | 13.3 | 13.6 | 10.5 | 14.2 |  | 9.5 | 9.8 | 9.3 | 8.2 | 9.9 |
| 56 | LSTc - 07 | Neu5Aca2-6Galb1-3GlcNAcb1-3Galb1-BSA | 9.4 | 11.7 | 10.6 | 7.6 | 12.7 |  | 7.3 | 8.6 | 7.4 | 7.2 | 7.4 |
| 57 | GA1 - 20 | Galb1-3GalNAcb1-4Galb1-BSA (GA1tri or asialo-GM1) | 13.9 | 14.6 | 14.0 | 11.8 | 14.4 |  | 11.0 | 10.6 | 12.6 | 9.5 | 11.0 |
| 58 | BG-B4-06 | Gala1-3(Fuca1-2)Galb1-3GalNAcb1-linker-BSA | 9.2 | 12.0 | 12.6 | 7.8 | 8.4 |  | 7.5 | 8.9 | 8.6 | 7.2 | 7.2 |
| 59 | BG-B4-High | Gala1-3(Fuca1-2)Galb1-3GalNAcb1-linker-BSA | 12.7 | 13.9 | 14.8 | 10.7 | 13.2 |  | 9.1 | 10.1 | 10.4 | 8.4 | 7.6 |
| 60 | BG-A3-04 | GalNAca1-3(Fuca1-2)Galb1-3GalNAcα1-linker-BSA | 12.1 | 8.0 | 9.6 | 7.2 | 10.8 |  | 9.1 | 7.2 | 7.2 | 7.2 | 7.2 |
| 61 | BG-A3-14 | GalNAca1-3(Fuca1-2)Galb1-3GalNAcα1-linker-BSA | 15.1 | 11.2 | 15.0 | 10.3 | 15.0 |  | 11.7 | 7.9 | 9.9 | 8.0 | 10.7 |
| 62 | BG-A4-05 | GalNAca1-3(Fuca1-2)Galb1-3GalNAcb1-linker-BSA | 13.0 | 7.9 | 10.1 | 7.2 | 12.7 |  | 9.7 | 7.2 | 7.2 | 7.2 | 7.2 |
| 63 | BG-A4-14 | GalNAca1-3(Fuca1-2)Galb1-3GalNAcb1-linker-BSA | 15.7 | 11.8 | 15.6 | 8.7 | 15.9 |  | 12.0 | 8.6 | 10.5 | 7.3 | 11.2 |
| 64 | BG-B2-03 | Gala1-3(Fuca1-2)Galb1-4GlcNAcb1-linker-BSA | 8.5 | 8.5 | 9.1 | 9.3 | 9.2 |  | 7.9 | 7.6 | 7.3 | 7.6 | 7.2 |
| 65 | BG-B3-05 | Gala1-3(Fuca1-2)Galb1-3GalNAcα1-linker-BSA | 7.2 | 8.2 | 11.1 | 7.2 | 7.2 |  | 7.2 | 7.5 | 7.2 | 7.2 | 7.2 |
| 66 | BG-B3-17 | Gala1-3(Fuca1-2)Galb1-3GalNAcα1-linker-BSA | 11.9 | 12.8 | 13.9 | 10.9 | 14.3 |  | 8.8 | 9.4 | 9.2 | 8.7 | 8.5 |
| 67 | BG-A1-04 | GalNAca1-3(Fuca1-2)Galb1-3GlcNAcb1-linker-BSA | 12.9 | 7.9 | 11.3 | 8.2 | 12.7 |  | 9.8 | 7.3 | 7.7 | 7.2 | 7.2 |
| 68 | BG-A1-12 | GalNAca1-3(Fuca1-2)Galb1-3GlcNAcb1-linker-BSA | 14.8 | 12.5 | 14.2 | 11.7 | 15.1 |  | 10.6 | 9.1 | 10.5 | 8.5 | 10.2 |
| 69 | BG-A2-04 | GalNAca1-3(Fuca1-2)Galb1-4GlcNAcb1-linker-BSA | 12.2 | 7.2 | 12.4 | 7.2 | 13.0 |  | 9.2 | 7.2 | 8.6 | 7.2 | 7.5 |
| 70 | BG-A2-16 | GalNAca1-3(Fuca1-2)Galb1-4GlcNAcb1-linker-BSA | 15.8 | 12.4 | 16.1 | 10.0 | 16.2 |  | 12.1 | 9.1 | 11.8 | 7.6 | 11.4 |
| 71 | BG-B1-05 | Gala1-3(Fuca1-2)Galb1-3GlcNAcb1-linker-BSA | 8.4 | 8.0 | 8.2 | 10.6 | 7.2 |  | 7.2 | 7.2 | 7.2 | 8.0 | 7.2 |
| 72 | BG-B1-15 | Gala1-3(Fuca1-2)Galb1-3GlcNAcb1-linker-BSA | 13.0 | 13.5 | 10.9 | 13.0 | 12.9 |  | 9.4 | 9.8 | 7.4 | 9.6 | 7.5 |
| 73 | BG-B2-17 | Gala1-3(Fuca1-2)Galb1-4GlcNAcb1-linker-BSA | 11.0 | 12.9 | 12.4 | 9.9 | 12.3 |  | 8.3 | 9.4 | 8.6 | 7.8 | 7.2 |
| 74 | Ac-Tn(Ser)-Tn(Ser)-Tn(Ser)-G - 16 | Ac-(GalNAca)Ser-(GalNAca)Ser-(GalNAca)Ser-Gly-Hex-BSA | 16.1 | 14.9 | 16.3 | 15.1 | 16.3 |  | 12.4 | 11.3 | 11.9 | 11.4 | 11.6 |
| 75 | Chitotriose - 08 | GlcNAcb1-4GlcNAcb1-4GlcNAcb-BSA | 15.4 | 15.7 | 16.1 | 14.3 | 15.4 |  | 12.2 | 11.9 | 11.3 | 10.6 | 11.3 |
| 76 | Gal3- 07 | Gala1-3Galb1-4Gala-BSA | 12.4 | 12.4 | 12.4 | 10.4 | 13.3 |  | 9.4 | 9.3 | 9.0 | 7.8 | 9.3 |
| 77 | Man3 - 05 | Mana1-6(Mana1-3)Manb1-4GlcNAc -BSA | 9.0 | 7.2 | 7.8 | 7.3 | 7.2 |  | 7.2 | 7.2 | 7.2 | 7.3 | 7.2 |
| 78 | Ac-S-S-S-G - 24 | Ac-Ser-Ser-Ser-Gly-Hex-BSA | 11.1 | 14.2 | 12.4 | 10.8 | 12.3 |  | 7.6 | 10.4 | 7.8 | 8.2 | 7.2 |
| 79 | Ac-S-Tn(Ser)-S-G - 22 | Ac-Ser-(GalNAca)Ser-Ser-Gly-Hex-BSA | 16.1 | 14.8 | 16.1 | 15.1 | 16.3 |  | 12.3 | 11.1 | 11.7 | 11.6 | 11.5 |
| 80 | P1 - 09 | Gala1-4Galb1-4GlcNAc-BSA | 12.7 | 11.6 | 14.1 | 10.7 | 14.0 |  | 9.6 | 7.8 | 9.7 | 8.3 | 8.8 |
| 81 | Gb4 - 09 | GalNAcb1-3Gala1-4Galb1-BSA (aka: P antigen) | 11.2 | 10.0 | 12.5 | 9.6 | 14.5 |  | 8.4 | 7.8 | 8.8 | 7.9 | 10.5 |
| 82 | GM1a - 29 | Galb1-3GalNAcb1-4(Neu5Aca2-3)Galb1-4(Glc)HSA | 10.6 | 12.3 | 12.4 | 11.9 | 13.1 |  | 8.6 | 9.4 | 9.6 | 9.3 | 9.3 |
| 83 | pLNH - 21 | Galb1-3GlcNAcb1-3Galb1-4GlcNAcb1-3Galb1-BSA | 13.8 | 14.2 | 14.4 | 12.4 | 15.3 |  | 9.8 | 10.1 | 10.2 | 8.9 | 9.5 |
| 84 | HSA (recomb) | human serum albumin (recombinant) | 10.2 | 9.5 | 9.5 | 8.3 | 7.8 |  | 7.7 | 7.3 | 7.4 | 7.2 | 7.2 |
| 85 | LNnT - 14 | Galb1-4GlcNAcb1-3Galb1-BSA | 11.5 | 13.7 | 14.1 | 14.2 | 13.5 |  | 8.9 | 9.2 | 9.9 | 11.1 | 7.5 |
| 86 | Man5 - 05 | Mana1-6(Mana1-3)Mana1-6(Mana1-3)Manb1-4GlcNAc-BSA | 7.5 | 7.4 | 8.3 | 7.6 | 7.2 |  | 7.2 | 7.2 | 7.2 | 7.2 | 7.2 |
| 87 | Gb4 tetra (P1 tetra)-Sp - 06 | GalNAcb1-3Gala1-4Galb1-4GlcNAcb-Sp-BSA | 11.4 | 9.9 | 14.3 | 9.9 | 14.3 |  | 9.6 | 7.8 | 10.5 | 9.0 | 11.3 |
| 88 | Man6 - II - 05 | Mana1-2Mana1-3Mana1-6(Mana1-2Mana1-3)Manb1-BSA | 12.2 | 7.2 | 7.7 | 12.5 | 7.2 |  | 9.4 | 7.2 | 7.2 | 9.4 | 7.2 |
| 89 | DFpLNH I, LeB - 09 | Fuca1-2Galb1-3(Fuca1-4)GlcNAcb1-3Galb1-4GlcNAcb1-3Galb-BSA | 10.2 | 10.0 | 10.9 | 9.1 | 7.6 |  | 7.5 | 7.6 | 7.4 | 7.2 | 7.2 |
| 90 | DSLNT - 06 | Neu5Aca2-3Galb1-3(Neu5Aca2-6)GlcNAcb1-3Galb1-BSA | 9.0 | 8.7 | 9.5 | 8.3 | 7.2 |  | 7.2 | 7.2 | 7.2 | 7.2 | 7.2 |
| 91 | Man9 #2 | Mana1-2Mana1-6(Mana1-2Mana1-3)Mana1-6(Mana1-2Mana1-2Mana1-3)Manb1-4GlcNAc-BSA | 10.3 | 10.2 | 10.5 | 9.0 | 10.7 |  | 8.3 | 8.1 | 8.1 | 7.4 | 7.2 |
| 92 | 2'F-B type 2-Sp - 07 | Gala1-3[Fuca1-2]Galb1-4[Fuca1-3]GlcNAcb-Sp-BSA | 9.3 | 8.1 | 8.1 | 7.2 | 7.2 |  | 7.2 | 7.2 | 7.2 | 7.2 | 7.2 |
| 93 | Man7D3 - 08 | Mana1-2Mana1-6(Mana1-3)Mana1-6(Mana1-2Mana1-3)Manb1-4GlcNAc-BSA | 10.1 | 7.7 | 9.6 | 8.6 | 9.5 |  | 7.6 | 7.2 | 7.2 | 7.2 | 7.2 |
| 94 | Globo A - 03 | GalNAca1-3(Fuca1-2)Galb1-3GalNAcb1-3Gala1-4Galb1-BSA | 12.1 | 7.9 | 11.0 | 8.7 | 12.5 |  | 9.5 | 7.3 | 7.5 | 7.3 | 7.3 |
| 95 | Man7D1 - 10 | Mana1-6(Mana1-3)Mana1-6(Mana1-2Mana1-2Mana1-3)Manb1-4GlcNAc-BSA | 10.1 | 9.0 | 10.0 | 7.9 | 10.1 |  | 7.9 | 7.4 | 7.6 | 7.2 | 7.2 |
| 96 | GM2-Sp - 14 | Neu5Aca2-3[GalNAcb1-4]Galb1-4Glcb-Sp-BSA | 10.9 | 12.1 | 13.7 | 12.6 | 14.3 |  | 8.4 | 8.9 | 9.5 | 9.1 | 9.9 |
| 98 | BG-H4- 04 | Fuca1-2Galb1-3GalNAcb1-linker-BSA | 12.5 | 10.5 | 11.7 | 9.2 | 10.7 |  | 9.1 | 8.2 | 8.3 | 7.9 | 7.2 |
| 99 | BG-A1- 05 | GalNAca1-3(Fuca1-2)Galb1-3GlcNAcb1-3Galb1-4(Glc)-APD-HSA | 13.7 | 11.7 | 14.2 | 9.3 | 13.7 |  | 10.4 | 9.0 | 10.1 | 8.2 | 9.6 |
| 100 | BG-H3- 04 | Fuca1-2Galb1-3GalNAcα1-linker-BSA | 8.4 | 8.9 | 9.1 | 7.2 | 8.7 |  | 7.5 | 7.6 | 7.4 | 7.2 | 7.2 |
| 101 | BG-B (Dextra) - 13 | Gala1-3(Fuca1-2)Galb-BSA [BG-B] from Dextra | 12.2 | 12.2 | 15.2 | 11.3 | 13.4 |  | 9.5 | 10.4 | 11.4 | 8.9 | 9.9 |
| 102 | alphaGal- 08 | Gala1-3Galb1-4GlcNAc-BSA | 12.5 | 12.5 | 12.4 | 9.0 | 13.8 |  | 9.5 | 9.1 | 9.4 | 7.6 | 10.5 |
| 103 | Cellotriose - 13 | Glcb1-4Glcb1-4Glcb-BSA | 15.7 | 16.3 | 15.8 | 15.5 | 16.0 |  | 11.8 | 12.3 | 11.3 | 10.9 | 11.0 |
| 104 | Maltopentaose - 11 | Glca1-4Glca1-4Glca1-4Glca1-4Glca-BSA | 12.8 | 14.4 | 13.3 | 13.2 | 13.7 |  | 9.7 | 11.5 | 9.3 | 9.2 | 9.6 |
| 105 | BG-H4- 15 | Fuca1-2Galb1-3GalNAcb1-linker-BSA | 14.8 | 13.6 | 15.4 | 12.3 | 14.7 |  | 11.2 | 10.2 | 11.1 | 9.4 | 10.0 |
| 106 | Globo B - 05 | Gala1-3(Fuca1-2)Galb1-3GalNAcb1-3Gala1-4Galb1-BSA | 10.1 | 9.9 | 10.4 | 9.1 | 11.2 |  | 7.9 | 8.2 | 7.8 | 7.5 | 7.6 |
| 107 | LacNAc (trimeric) - 08 | Galb1-4GlcNAcb1-3Galb1-4GlcNAcb1-3Galb1-4GlcNAcb-APE-HSA | 11.1 | 11.4 | 12.1 | 9.6 | 12.3 |  | 8.7 | 8.3 | 10.5 | 7.5 | 7.4 |
| 108 | Forssman Di - 21 | GalNAca1-3GalNAcb1-BSA | 15.8 | 16.3 | 16.4 | 15.9 | 16.3 |  | 16.1 | 12.2 | 12.0 | 12.4 | 11.7 |
| 109 | Ac-S-TF(Ser)-S-G - 16 | Ac-Ser-(Galb1-3GalNAca)Ser-Ser-Gly-Hex-BSA | 14.7 | 14.8 | 15.1 | 14.7 | 15.9 |  | 11.9 | 10.9 | 10.6 | 11.5 | 10.9 |
| 110 | Globo H - 03 | Fuca1-2Galb1-3GalNAcb1-3Gala1-4Galb1-BSA | 10.9 | 10.3 | 11.2 | 9.5 | 12.1 |  | 9.6 | 7.7 | 8.0 | 7.8 | 7.2 |
| 111 | LeC-Sp - 06 | Galb1-3GlcNAcb-Sp-BSA | 12.8 | 13.1 | 13.0 | 11.0 | 14.6 |  | 10.0 | 9.3 | 9.0 | 8.3 | 8.9 |
| 112 | Globo B - 12 | Gala1-3(Fuca1-2)Galb1-3GalNAcb1-3Gala1-4Galb1-BSA | 9.9 | 10.8 | 12.8 | 9.1 | 12.8 |  | 8.2 | 8.3 | 8.7 | 7.4 | 8.3 |
| 113 | GalNAc-a - 04 | GalNAc-a - BSA | 15.4 | 14.6 | 14.8 | 14.1 | 15.0 |  | 11.9 | 11.1 | 10.5 | 10.8 | 10.4 |
| 114 | GalNAca1-6Galb - 04 | GalNAca1-6Galb-BSA | 15.0 | 13.6 | 14.9 | 12.8 | 15.0 |  | 12.0 | 10.0 | 10.5 | 10.2 | 10.5 |
| 115 | Adi - 04 | GalNAca1-3Galb-BSA | 15.1 | 14.4 | 16.1 | 13.2 | 16.2 |  | 11.7 | 10.7 | 12.2 | 10.4 | 11.8 |
| 116 | GA2di - 05 | GalNAcb1-4Galb - BSA (aka: asialo-GM2) | 13.0 | 14.9 | 14.4 | 12.8 | 14.2 |  | 10.4 | 11.1 | 11.6 | 12.6 | 9.4 |
| 117 | LNT - 05 | Galb1-3GlcNAcb1-3Galb-BSA | 10.6 | 11.0 | 12.0 | 9.2 | 12.6 |  | 9.0 | 8.8 | 8.3 | 7.3 | 8.9 |
| 118 | GA1 - 06 | Galb1-3GalNAcb1-4Galb1-BSA (GA1tri or asialo-GM1) | 13.2 | 12.8 | 12.9 | 10.9 | 13.2 |  | 9.9 | 9.7 | 11.3 | 8.6 | 9.4 |
| 119 | Forssman Di - 04 | GalNAca1-3GalNAcb1-BSA | 14.9 | 15.5 | 16.2 | 14.7 | 16.2 |  | 15.1 | 11.5 | 11.8 | 11.4 | 11.5 |
| 120 | Ac-S-Tn(Ser)-S-G - 04 | AcSer-(GalNAca)Ser-Ser-Gly-Hex-BSA | 13.8 | 12.1 | 14.1 | 11.4 | 15.9 |  | 10.2 | 9.1 | 9.8 | 8.8 | 11.1 |
| 121 | Ac-Tn(Ser)Tn(Ser)Tn(Ser)-G - 03 | Ac-(GalNAca)Ser-(GalNAca)Ser-(GalNAca)Ser-Gly-Hex-BSA | 13.0 | 12.0 | 14.4 | 11.0 | 13.9 |  | 10.4 | 9.1 | 9.8 | 8.3 | 10.5 |
| 122 | Ac-S-Tn(Thr)-S-G - 04 | Ac-Ser-(GalNAca)Thr-Ser-Gly-Hex-BSA | 13.0 | 10.8 | 13.0 | 9.9 | 13.4 |  | 10.2 | 8.5 | 9.1 | 8.1 | 9.5 |
| 123 | Ac-S-TF(Ser)-S-G - 04 | Ac-Ser-(Galb1-3GalNAca)Ser-Ser-Gly-Hex-BSA | 11.3 | 12.0 | 10.6 | 10.6 | 13.2 |  | 8.7 | 9.0 | 7.3 | 8.6 | 7.9 |
| 124 | GA2di - 37 | GalNAcb1-4Galb - BSA (aka: asialo-GM2) | 15.1 | 15.8 | 16.0 | 14.5 | 15.8 |  | 12.1 | 11.9 | 12.3 | 15.1 | 10.6 |
| 125 | Forssman Di - 31 | GalNAca1-3GalNAcb1-BSA | 15.7 | 16.3 | 16.4 | 16.1 | 16.2 |  | 16.2 | 12.2 | 12.0 | 12.6 | 11.6 |
| 126 | Ac-S-Tn(Ser)-S-G - 33 | Ac-Ser-(GalNAca)Ser-Ser-Gly-Hex-BSA | 16.2 | 15.3 | 16.2 | 15.4 | 16.3 |  | 13.0 | 11.5 | 11.9 | 12.0 | 11.5 |
| 127 | Ac-Tn(Ser)Tn(Ser)Tn(Ser)-G - 27 | Ac-(GalNAca)Ser-(GalNAca)Ser-(GalNAca)Ser-Gly-Hex-BSA | 15.9 | 15.4 | 16.3 | 15.6 | 16.1 |  | 12.7 | 11.6 | 12.1 | 12.0 | 11.5 |
| 128 | Ac-S-TF(Ser)-S-G - 28 | Ac-Ser-(Galb1-3GalNAca)Ser-Ser-Gly-Hex-BSA | 15.2 | 15.1 | 15.3 | 14.8 | 16.1 |  | 11.4 | 11.3 | 11.3 | 11.9 | 11.4 |
| 129 | Chitotriose - 20 | GlcNAcb1-4GlcNAcb1-4GlcNAcb-BSA | 16.1 | 16.2 | 16.4 | 15.6 | 16.0 |  | 12.7 | 12.3 | 12.1 | 12.1 | 11.6 |
| 130 | Fuc-a - 04 | Fuc-a - BSA | 14.1 | 14.6 | 13.2 | 12.8 | 14.3 |  | 10.5 | 10.6 | 9.4 | 9.9 | 9.8 |
| 131 | Fuc-b - 04 | Fuc-b - BSA | 12.8 | 14.0 | 12.2 | 9.3 | 14.7 |  | 9.6 | 8.9 | 8.7 | 8.2 | 8.1 |
| 132 | GlcNAca1-4Galb - 03 | GlcNAca1-4Galb-BSA | 11.8 | 13.2 | 12.0 | 11.4 | 12.7 |  | 9.0 | 9.4 | 7.7 | 8.6 | 8.5 |
| 133 | LNH - 13 | Galb1-4GlcNAcb1-6(Galb1-3GlcNAcb1-3)Galb1-BSA | 12.0 | 12.3 | 13.3 | 10.3 | 13.7 |  | 9.3 | 9.0 | 8.9 | 7.9 | 9.2 |
| 134 | LNnH - 11 | Galb1-4GlcNAcb1-6(Galb1-4GlcNAcb1-3)Galb1-BSA | 11.1 | 10.0 | 10.6 | 8.8 | 11.7 |  | 8.4 | 7.4 | 7.3 | 7.5 | 7.2 |
| 135 | LNnT - 04 | Galb1-4GlcNAcb1-3Galb1-BSA | 10.4 | 9.2 | 10.2 | 8.7 | 8.3 |  | 8.4 | 7.5 | 7.4 | 7.4 | 7.2 |
| 136 | pLNH - 07 | Galb1-3GlcNAcb1-3Galb1-4GlcNAcb1-3Galb1-BSA | 11.6 | 11.4 | 12.0 | 9.4 | 13.0 |  | 8.6 | 8.0 | 7.8 | 7.5 | 7.3 |
| 137 | BG-H1- 06 | Fuca1-2Galb1-3GlcNAcb1-linker-BSA | 13.3 | 11.7 | 10.5 | 9.8 | 8.6 |  | 9.6 | 8.6 | 7.3 | 8.1 | 7.2 |
| 138 | BG-H2- 06 | Fuca1-2Galb1-4GlcNAcb1-linker-BSA | 11.5 | 10.5 | 11.0 | 9.8 | 11.7 |  | 9.2 | 8.0 | 7.9 | 8.0 | 7.3 |
| 139 | BG-H2- 12 | Fuca1-2Galb1-4GlcNAcb1-linker-BSA | 11.4 | 12.4 | 12.7 | 10.1 | 12.7 |  | 8.8 | 9.2 | 8.6 | 8.0 | 7.2 |
| 140 | BG-H3- 14 | Fuca1-2Galb1-3GalNAcα1-linker-BSA | 13.1 | 13.6 | 14.9 | 12.1 | 15.1 |  | 9.8 | 10.0 | 10.5 | 9.2 | 9.8 |
| 141 | Ac-S-Tn(Thr)-A-G - 04 | Ac-Ser-(GalNAca)Thr-Ala-Gly-Hex-BSA (muc1) | 13.7 | 13.1 | 14.3 | 11.2 | 14.8 |  | 10.7 | 9.4 | 9.8 | 8.8 | 9.6 |
| 142 | Ac-S-Tn(Thr)-A-G - 08 | Ac-Ser-(GalNAca)Thr-Ala-Gly-Hex-BSA (muc1) | 15.0 | 14.3 | 15.4 | 12.7 | 15.7 |  | 11.7 | 10.5 | 11.1 | 9.9 | 10.9 |
| 143 | Ac-S-Tn(Thr)-A-G - 22 | Ac-Ser-(GalNAca)Thr-Ala-Gly-Hex-BSA (muc1) | 16.0 | 15.3 | 16.0 | 14.0 | 16.2 |  | 12.4 | 11.5 | 11.9 | 10.8 | 11.5 |
| 144 | Ac-V-Tn(Thr)-S-G - 04 | Ac-Val-(GalNAca)Thr-Ser-Gly-Hex-BSA (muc1) | 12.6 | 10.3 | 13.7 | 9.6 | 14.9 |  | 9.7 | 7.9 | 9.8 | 8.1 | 10.2 |
| 145 | Ac-V-Tn(Thr)-S-G - 08 | Ac-Val-(GalNAca)Thr-Ser-Gly-Hex-BSA (muc1) | 14.4 | 12.7 | 14.7 | 11.4 | 15.7 |  | 11.0 | 9.1 | 10.7 | 9.3 | 11.0 |
| 146 | Ac-V-Tn(Thr)-S-G - 19 | Ac-Val-(GalNAca)Thr-Ser-Gly-Hex-BSA (muc1) | 15.7 | 14.0 | 15.5 | 13.0 | 16.2 |  | 11.9 | 10.2 | 11.3 | 10.1 | 11.5 |
| 147 | Ac-A-Tn(Thr)-S-G - 05 | Ac-Ala-(GalNAca)Thr-Ser-Gly-Hex-BSA (muc4) | 13.8 | 12.3 | 14.3 | 10.9 | 15.0 |  | 10.5 | 9.2 | 10.2 | 9.0 | 10.3 |
| 148 | Ac-A-Tn(Thr)-S-G - 08 | Ac-Ala-(GalNAca)Thr-Ser-Gly-Hex-BSA (muc4) | 15.1 | 13.6 | 15.1 | 12.5 | 15.8 |  | 11.4 | 10.0 | 10.7 | 9.8 | 11.0 |
| 149 | Ac-A-Tn(Thr)-S-G - 23 | Ac-Ala-(GalNAca)Thr-Ser-Gly-Hex-BSA (muc4) | 16.0 | 14.5 | 15.8 | 13.7 | 16.2 |  | 12.3 | 10.9 | 11.6 | 10.7 | 11.4 |
| 150 | Ac-S-Tn(Thr)-G-G - 03 | Ac-Ser-(GalNAca)Thr-Gly-Gly-Hex-BSA (muc4) | 14.1 | 12.7 | 14.5 | 11.7 | 16.0 |  | 10.6 | 9.0 | 10.2 | 9.5 | 11.1 |
| 151 | Ac-S-Tn(Thr)-G-G - 07 | Ac-Ser-(GalNAca)Thr-Gly-Gly-Hex-BSA (muc4) | 15.4 | 13.7 | 15.3 | 13.1 | 16.2 |  | 11.4 | 9.9 | 10.7 | 10.2 | 11.2 |
| 152 | Ac-S-Tn(Thr)-G-G - 19 | Ac-Ser-(GalNAca)Thr-Gly-Gly-Hex-BSA (muc4) | 16.1 | 14.9 | 16.1 | 14.5 | 16.2 |  | 12.2 | 11.1 | 11.4 | 11.2 | 11.3 |
| 153 | Ac-T-Tn(Thr)-P-G - 04 | Ac-Thr-(GalNAca)Thr-Pro-Gly-Hex-BSA (muc2,6,7) | 14.5 | 12.3 | 14.4 | 12.0 | 15.4 |  | 10.7 | 8.9 | 10.1 | 9.6 | 10.0 |
| 154 | Ac-T-Tn(Thr)-P-G - 08 | Ac-Thr-(GalNAca)Thr-Pro-Gly-Hex-BSA (muc2,6,7) | 15.2 | 13.7 | 15.2 | 13.3 | 15.8 |  | 11.2 | 9.6 | 10.6 | 10.2 | 10.6 |
| 155 | Ac-T-Tn(Thr)-P-G - 21 | Ac-Thr-(GalNAca)Thr-Pro-Gly-Hex-BSA (muc2,6,7) | 15.9 | 14.7 | 15.9 | 14.5 | 16.1 |  | 11.9 | 10.6 | 11.2 | 11.0 | 11.1 |
| 156 | Ac-S-Tn(Thr)-S-G HSA-23 | Ac-Ser-(GalNAca)Thr-Ser-Gly-Hex-HSA | 16.1 | 14.7 | 16.0 | 13.9 | 16.2 |  | 12.3 | 11.0 | 11.7 | 10.6 | 11.4 |
| 157 | Ac-S-Tn(Thr)-S-G HSA-04 | Ac-Ser-(GalNAca)Thr-Ser-Gly-Hex-HSA | 13.5 | 11.8 | 14.0 | 10.8 | 14.9 |  | 10.6 | 8.5 | 10.0 | 8.7 | 10.1 |
| 158 | Ac-TF(Ser)-G - 04 | Ac-(Galb1-3GalNAca)Ser-Gly-Hex-BSA | 14.7 | 13.7 | 13.6 | 13.3 | 15.1 |  | 10.4 | 10.1 | 9.5 | 10.3 | 10.5 |
| 159 | Ac-TF(Ser)-G - 24 | Ac-(Galb1-3GalNAca)Ser-Gly-Hex-BSA | 16.3 | 15.5 | 15.9 | 15.2 | 16.2 |  | 11.6 | 11.7 | 11.8 | 11.9 | 11.5 |
| 160 | NA2 - 08 | Galb1-4GlcNAcb1-2Mana1-6[Galb1-4GlcNAcb1-2Mana1-3]Manb1-4GlcNAc -BSA | 9.9 | 9.7 | 10.1 | 8.6 | 10.1 |  | 7.8 | 7.7 | 7.3 | 7.3 | 7.2 |
| 161 | NA3 - 05 | Galb1-4GlcNAcb1-2Mana1-6[Galb1-4GlcNAcb1-2(Galb1-4GlcNAcb1-4)Mana1-3]Manb1-4GlcNAc -BSA | 7.4 | 7.2 | 7.2 | 7.2 | 7.2 |  | 7.2 | 7.2 | 7.2 | 7.2 | 7.2 |
| 162 | NGA2 - 07 | GlcNAcb1-2Mana1-6(GlcNAcb1-2Mana1-3)Manb1-4GlcNAc -BSA | 10.7 | 10.2 | 10.2 | 8.7 | 10.5 |  | 8.3 | 7.6 | 7.2 | 7.4 | 7.2 |
| 163 | NGA2B - 05 | GlcNAcb1-2Mana1-6(GlcNAcb1-2Mana1-3)(GlcNAcb1-4)Manb1-4GlcNAc -BSA | 9.3 | 7.2 | 8.1 | 7.7 | 7.8 |  | 7.7 | 7.2 | 7.2 | 7.3 | 7.2 |
| 164 | NA4 - 05 | Galb1-4GlcNAcb1-2(Galb1-4GlcNAcb1-6)Mana1-6[Galb1-4GlcNAcb1-2(Galb1-4GlcNAcb1-4)Mana1-3]Manb1-4GlcNAc -BSA | 10.0 | 9.1 | 9.7 | 7.6 | 9.8 |  | 7.5 | 7.3 | 7.4 | 7.2 | 7.2 |
| 165 | NGA3B - 06 | GlcNAcb1-2Mana1-6[GlcNAcb1-2(GlcNAcb1-4)Mana1-3](GlcNAcb1-4)Manb1-4GlcNAc -BSA | 11.3 | 12.8 | 10.6 | 9.6 | 11.8 |  | 8.3 | 8.7 | 7.6 | 7.4 | 7.5 |
| 166 | NGA3 - 01 | GlcNAcb1-2Mana1-6[GlcNAcb1-2(GlcNAcb1-4)Mana1-3]Manb1-4GlcNAc -BSA | 10.9 | 10.2 | 9.9 | 9.0 | 10.0 |  | 8.4 | 7.8 | 7.2 | 7.5 | 7.2 |
| 167 | NGA4 - 06 | GlcNAcb1-2(GlcNAcb1-6)Mana1-6[GlcNAcb1-2(GlcNAcb1-4)Mana1-3]Manb1-4GlcNAc -BSA | 11.8 | 13.0 | 11.5 | 10.3 | 12.0 |  | 8.8 | 9.1 | 7.8 | 7.9 | 7.2 |
| 168 | NGA4(B)2 - 04 | GlcNAcb1-2(GlcNAcb1-4)(GlcNAcb1-6)Mana1-6[GlcNAcb1-2Mana1-3](GlcNAcb1-4)Manb1-4GlcNAc -BSA | 11.4 | 13.4 | 12.2 | 10.3 | 12.2 |  | 8.6 | 9.5 | 7.5 | 7.8 | 7.2 |
| 169 | NGA5B - 02 | GlcNAcb1-2(GlcNAcb1-4)(GlcNAcb1-6)Mana1-6[GlcNAcb1-2(GlcNAcb1-4)Mana1-3](GlcNAcb1-4)Manb1-4GlcNAc -BSA | 11.6 | 13.2 | 11.4 | 9.9 | 13.1 |  | 8.8 | 9.4 | 7.6 | 7.9 | 7.4 |
| 170 | GA2di (accurate) - 28 | GalNAcb1-4Galb - BSA (aka: asialo-GM2) | 15.2 | 15.6 | 15.7 | 14.5 | 15.8 |  | 11.4 | 11.6 | 12.4 | 15.1 | 10.4 |
| 171 | Ac-P-Tn(Thr)-T-G - 05 | Ac-Pro-(GalNAca)Thr-Thr-Gly-Hex-BSA (muc2) | 14.9 | 13.1 | 14.5 | 11.7 | 15.3 |  | 11.4 | 9.4 | 11.0 | 11.0 | 10.6 |
| 172 | Ac-P-Tn(Thr)-T-G - 08 | Ac-Pro-(GalNAca)Thr-Thr-Gly-Hex-BSA (muc2) | 15.7 | 13.9 | 15.3 | 12.9 | 16.0 |  | 12.1 | 10.0 | 10.9 | 10.5 | 11.3 |
| 173 | Ac-P-Tn(Thr)-T-G - 22 | Ac-Pro-(GalNAca)Thr-Thr-Gly-Hex-BSA (muc2) | 16.2 | 15.1 | 15.9 | 13.7 | 16.2 |  | 12.6 | 11.0 | 11.9 | 10.8 | 11.6 |
| 174 | Ac-Tn(Thr)-Tn(Thr)-Tn(Thr)-G - 05 | Ac-(GalNAca)Thr-(GalNAca)Thr-(GalNAca)Thr-Gly-Hex-BSA (muc2) | 15.3 | 12.8 | 14.4 | 12.3 | 15.2 |  | 11.5 | 9.4 | 10.2 | 9.5 | 10.8 |
| 175 | Ac-Tn(Thr)-Tn(Thr)-Tn(Thr)-G - 08 | Ac-(GalNAca)Thr-(GalNAca)Thr-(GalNAca)Thr-Gly-Hex-BSA (muc2) | 15.6 | 13.3 | 15.1 | 13.3 | 15.6 |  | 11.9 | 9.7 | 10.7 | 9.9 | 11.4 |
| 176 | Ac-Tn(Thr)-Tn(Thr)-Tn(Thr)-G - 20 | Ac-(GalNAca)Thr-(GalNAca)Thr-(GalNAca)Thr-Gly-Hex-BSA (muc2) | 16.2 | 14.6 | 15.8 | 14.3 | 16.2 |  | 12.4 | 10.4 | 11.5 | 11.0 | 11.5 |
| 177 | Ac-S-Tn(Thr)-Tn(Thr)-G - 05 | Ac-Ser-(GalNAca)Thr-(GalNAca)Thr-Gly-Hex-BSA (muc2) | 15.9 | 12.2 | 15.0 | 11.8 | 16.1 |  | 12.0 | 8.6 | 10.7 | 9.3 | 11.3 |
| 178 | Ac-S-Tn(Thr)-Tn(Thr)-G - 09 | Ac-Ser-(GalNAca)Thr-(GalNAca)Thr-Gly-Hex-BSA (muc2) | 16.1 | 13.4 | 15.3 | 13.0 | 16.0 |  | 12.3 | 9.4 | 11.0 | 10.0 | 11.4 |
| 179 | Ac-S-Tn(Thr)-Tn(Thr)-G - 22 | Ac-Ser-(GalNAca)Thr-(GalNAca)Thr-Gly-Hex-BSA (muc2) | 16.3 | 14.4 | 16.1 | 14.3 | 16.3 |  | 12.6 | 10.4 | 11.6 | 10.9 | 11.5 |
| 180 | Gala3-type1 - 09 | Gala1-3Galb1-3GlcNAc-BSA | 14.8 | 15.8 | 14.7 | 11.8 | 16.0 |  | 11.9 | 11.3 | 10.5 | 9.3 | 11.2 |
| 181 | 2'FucLac (BG-H6) - 07 | Fuca1-2Galb1-4Glc-BSA | 13.0 | 12.3 | 12.2 | 11.8 | 13.3 |  | 11.1 | 8.5 | 8.1 | 8.3 | 7.2 |
| 182 | Ac-S-Tn(Thr)-V-G - 04 | Ac-Ser-(GalNAca)Thr-Val-Gly-Hex-BSA | 13.7 | 12.6 | 14.1 | 11.0 | 14.8 |  | 10.3 | 8.8 | 9.5 | 8.2 | 9.0 |
| 183 | Ac-S-Tn(Thr)-V-G - 22 | Ac-Ser-(GalNAca)Thr-Val-Gly-Hex-BSA | 15.0 | 14.2 | 15.1 | 12.5 | 15.7 |  | 11.3 | 10.2 | 10.5 | 9.3 | 10.4 |
| 184 | Ac-G-V-Tn(Thr)-S-A-G - 04 | Ac-Gly-Val-(GalNAca)Thr-Ser-Ala-Gly-Hex-BSA (muc1) | 13.2 | 10.4 | 16.3 | 9.3 | 14.9 |  | 10.0 | 7.5 | 11.4 | 7.8 | 9.6 |
| 185 | Ac-G-V-Tn(Thr)-S-A-G - 21 | Ac-Gly-Val-(GalNAca)Thr-Ser-Ala-Gly-Hex-BSA (muc1) | 15.1 | 13.4 | 16.5 | 12.0 | 15.8 |  | 11.5 | 9.3 | 11.8 | 9.3 | 10.9 |
| 186 | GTSSA-TF(Ser)-TGHATPLPVTD | BSA-PEG7-Gly-Thr-Ser-Ser-Ala-(Galb1-3GalNAca)Ser-Thr-Gly-His-Ala-Thr-Pro-Leu-Pro-Val-Thr-Asp | 11.5 | 8.8 | 11.4 | 8.4 | 11.5 |  | 8.7 | 7.2 | 8.2 | 7.5 | 7.2 |
| 187 | GTSSAS-TF(Thr)-GHATPLPVTD | BSA-PEG7-Gly-Thr-Ser-Ser-Ala-Ser-(Galb1-3GalNAca)Thr-Gly-His-Ala-Thr-Pro-Leu-Pro-Val-Thr-Asp | 10.6 | 9.0 | 10.4 | 8.4 | 9.6 |  | 8.4 | 7.3 | 7.9 | 7.5 | 7.2 |
| 188 | GTSSASTGHA-TF(Thr)-PLPVTD | BSA-PEG7-Gly-Thr-Ser-Ser-Ala-Ser-Thr-Gly-His-Ala-(Galb1-3GalNAca)Thr-Pro-Leu-Pro-Val-Thr-Asp | 7.2 | 7.2 | 9.3 | 7.2 | 7.2 |  | 7.2 | 7.2 | 7.3 | 7.2 | 7.2 |
| 189 | GTSSA-TF(Ser)-TF(Thr)-GHATPLPVTD | BSA-PEG7-Gly-Thr-Ser-Ser-Ala-(Galb1-3GalNAca)Ser-(Galb1-3GalNAca)Thr-Gly-His-Ala-Thr-Pro-Leu-Pro-Val-Thr-Asp | 8.8 | 8.2 | 10.1 | 7.2 | 10.8 |  | 7.4 | 7.3 | 7.3 | 7.2 | 7.2 |
| 190 | GTSSASTGHATPLPVTD | BSA-PEG7-Gly-Thr-Ser-Ser-Ala-Ser-Thr-Gly-His-Ala-Thr-Pro-Leu-Pro-Val-Thr-Asp | 10.1 | 8.5 | 9.6 | 7.2 | 8.9 |  | 7.5 | 7.2 | 7.3 | 7.3 | 7.2 |
| 191 | PEG-linker - 06 | OH-(CH2)2-NH-Gly-CO-PEG7-NH-(CO)Hept-SH-Mal-Cychex-CO-BSA | 9.1 | 7.2 | 9.2 | 7.2 | 8.0 |  | 7.6 | 7.2 | 7.2 | 7.2 | 7.2 |
| 192 | Galilli - 21 | Gala1-3Galb1-4Glc-BSA | 15.5 | 16.2 | 15.9 | 13.5 | 16.2 |  | 11.8 | 11.9 | 11.7 | 10.0 | 11.6 |
| 193 | 6'Neu5Ac-LNF V - 12 | Fuca1-2Galb1-3(Neu5Aca2-6)GlcNAcb1-3Galb1-APD-HSA | 12.3 | 10.5 | 12.5 | 9.6 | 11.2 |  | 9.4 | 7.9 | 8.8 | 7.4 | 7.4 |
| 194 | Fuc, 6'Neu5Ac-LNnH-APD-HSA - 12 | Galb1-4[Fuca1-3]GlcNAcb1-6[Neu5Aca2-6Galb1-4GlcNAcb1-3]Galb1-APD-HSA | 10.3 | 11.7 | 10.9 | 8.6 | 12.4 |  | 7.3 | 8.3 | 7.2 | 7.2 | 7.5 |
| 195 | 3'Neu5Ac-3-FL - 07 | Neu5Aca2-3Galb1-4(Fuca1-3)Glc -BSA | 8.4 | 8.3 | 11.5 | 7.8 | 10.6 |  | 7.3 | 7.3 | 7.4 | 7.3 | 7.2 |
| 196 | GlcNAc-Man5 - 03 | Mana1-6(Mana1-3)Mana1-6(GlcNAcb1-2Mana1-3)Manb1-4GlcNAcb-BSA | 10.0 | 9.4 | 10.4 | 8.9 | 11.2 |  | 8.1 | 7.7 | 7.2 | 7.2 | 7.4 |
| 197 | GlcNAc-Man3 - 02 | Mana1-6(GlcNAcb1-2Mana1-3)Manb1-4GlcNAcb-BSA | 10.4 | 8.5 | 10.0 | 8.9 | 10.5 |  | 7.9 | 7.4 | 7.3 | 7.5 | 7.3 |
| 198 | LacNAc-Man5 - 02 | Mana1-6(Mana1-3)Mana1-6(Galb1-4GlcNAcb1-2Mana1-3)Manb1-4GlcNAcb-BSA | 10.1 | 10.3 | 10.7 | 9.0 | 11.2 |  | 8.6 | 8.2 | 7.9 | 7.5 | 7.3 |
| 199 | Ac-S-Thr-S-G - 18 | Ac-Ser-Thr-Ser-Gly-Hex-BSA | 11.1 | 13.0 | 12.5 | 9.2 | 11.1 |  | 8.4 | 9.1 | 8.4 | 7.8 | 7.2 |
| 200 | LeA-LeX - 21 | Galb1-3(Fuca1-4)GlcNAcb1-3Galb1-4(Fuca1-3)GlcNAcb1-3Galb1-APD-HSA | 11.0 | 8.4 | 12.3 | 8.4 | 12.5 |  | 8.4 | 7.5 | 8.5 | 7.3 | 7.4 |
| 201 | 3'Neu5Ac-LNnT - 09 | Neu5Aca2-3Galb1-4GlcNAcb1-3Galb1-APD-HSA | 11.4 | 10.5 | 8.7 | 8.0 | 12.0 |  | 9.3 | 8.0 | 7.2 | 7.6 | 7.2 |
| 202 | Hep-N-acetylated | fully N-acetylated heparin polysaccharide | 9.5 | 7.5 | 8.4 | 7.2 | 7.2 |  | 7.4 | 7.2 | 7.2 | 7.2 | 7.2 |
| 203 | Hep-5000 - 01 | heparin polysaccharide (MW ~5000) | 10.0 | 8.7 | 9.1 | 7.8 | 7.6 |  | 7.7 | 7.2 | 7.4 | 7.3 | 7.2 |
| 204 | Man6 - I - 04 | Mana1-6(Mana1-3)Mana1-6(Mana1-2Mana1-3)Manb1-BSA | 7.2 | 7.2 | 7.2 | 7.2 | 7.2 |  | 7.2 | 7.2 | 7.2 | 7.2 | 7.2 |
| 205 | 3'-sulpho-LeX - 15 | 3-SO3-Galb1-4[Fuca1-3)GlcNAc-BSA | 11.7 | 10.6 | 11.2 | 8.9 | 14.0 |  | 8.8 | 9.1 | 7.5 | 7.4 | 9.7 |
| 206 | Hya9 - 03 | (GlcAb1-3GlcNAcb1-4)4b1-3GlcAb1-BSA | 13.8 | 13.4 | 15.1 | 13.0 | 15.8 |  | 11.1 | 10.2 | 9.6 | 9.2 | 11.8 |
| 207 | 3'-sulpho-LeA - 15 | 3-SO3-Galb1-3[Fuca1-4)GlcNAc-BSA | 10.6 | 7.4 | 11.0 | 11.4 | 12.4 |  | 8.0 | 7.2 | 7.2 | 8.7 | 7.2 |
| 208 | Gala1-2Gal - 13 | Gala1-2Gal-BSA | 15.9 | 14.7 | 14.7 | 14.4 | 15.1 |  | 12.2 | 10.5 | 9.8 | 11.0 | 10.6 |
| 209 | 6'-sulpho-LeX - 08 | 6-SO3-Galb1-4[Fuca1-3)GlcNAc-BSA | 12.4 | 11.5 | 11.6 | 10.3 | 14.1 |  | 9.2 | 8.2 | 7.3 | 8.3 | 7.7 |
| 210 | Hya8 - 03 | (GlcNAcb1-4GlcAb1-3)4b1-BSA | 15.1 | 14.7 | 14.6 | 14.6 | 14.7 |  | 12.7 | 13.4 | 11.3 | 11.3 | 14.1 |
| 211 | 6'-sulpho-LeA - 16 | 6-SO3-Galb1-3[Fuca1-4)GlcNAc-BSA | 13.7 | 12.4 | 15.3 | 12.5 | 13.8 |  | 10.5 | 9.2 | 10.8 | 9.2 | 7.9 |
| 212 | MSMFLNH I - 11 | Neu5Aca2-6Galb1-4GlcNAcb1-6 (Fuca1-2Galb1-3GlcNAcb1-3)Galb1-BSA | 12.4 | 13.7 | 10.9 | 10.4 | 13.6 |  | 8.8 | 9.6 | 7.2 | 7.8 | 7.2 |
| 213 | A-LeB hexa - 06 | GalNAca1-3(Fuca1-2)Galb1-3(Fuca1-4)GlcNAcb1-3Galb1-BSA | 12.0 | 10.0 | 11.9 | 8.4 | 12.5 |  | 9.9 | 8.0 | 8.2 | 7.2 | 8.3 |
| 214 | Hybrid-M5N4B - 03 | GlcNAcb1-2Mana1-3[Mana1-3(Mana1-6)Mana1-6](GlcNAcb1-4)Manb1-4GlcNAcb1-BSA | 10.1 | 8.3 | 10.0 | 8.4 | 7.2 |  | 7.9 | 7.4 | 7.2 | 7.3 | 7.2 |
| 215 | MSMFLnNH - 09 | Galb1-4(Fuca1-3)GlcNAcb1-6 (Neu5Aca2-6Galb1-4GlcNAcb1-3)Galb1-BSA | 14.2 | 10.5 | 11.6 | 9.2 | 7.2 |  | 10.3 | 7.3 | 7.5 | 7.3 | 7.2 |
| 216 | iLNO - 06 | Galb1-3GlcNAcb1-3Galb1-4GlcNAcb1-6 (Galb1-3GlcNAcb1-3)Galb1-BSA | 11.2 | 10.8 | 12.1 | 9.2 | 12.8 |  | 8.7 | 7.8 | 8.5 | 7.3 | 7.6 |
| 217 | MFLNH I - 11 | Galb1-4GlcNAcb1-6 (Fuca1-2Galb1-3GlcNAcb1-3)Galb1-BSA | 12.7 | 10.3 | 11.2 | 8.1 | 8.7 |  | 9.7 | 7.7 | 8.0 | 7.2 | 7.2 |
| 218 | alphaGal-6-deoxy - 11 | Gala1-3Galb1-4(6deoxy-GlcNAc)-HSA (alphaGal)-HSA | 14.3 | 14.7 | 14.7 | 11.0 | 15.9 |  | 11.1 | 11.2 | 10.8 | 8.5 | 12.2 |
| 219 | MFLNH III - 14 | Galb1-4(Fuca1-3)GlcNAcb1-6 (Galb1-3GlcNAcb1-3)Galb1-BSA | 12.5 | 13.6 | 13.0 | 10.6 | 14.0 |  | 9.5 | 10.4 | 8.8 | 7.9 | 9.6 |
| 220 | TFiLNO(1-2,1-2,1-3) - 04 | Fuca1-2Galb1-3GlcNAcb1-3Galb1-4(Fuca1-3)GlcNAcb1-6(Fuca1-2Galb1-3GlcNAcb1-3)Galb-BSA (mixed with other stuff by MS) | 9.5 | 8.9 | 10.2 | 7.2 | 8.4 |  | 7.6 | 7.9 | 7.2 | 7.2 | 7.2 |
| 221 | LeC (dimeric)-Sp - 16 | Galb1-3GlcNAcb1-3Galb1-3GlcNAcb-Sp-BSA | 12.8 | 13.1 | 13.6 | 10.6 | 14.6 |  | 9.8 | 9.4 | 9.2 | 8.1 | 10.3 |
| 222 | 3'Neu5Ac(9Ac)-LacNAc-Sp - 04 | Neu5Ac(9Ac)a2-3Galb1-4GlcNAcb-Sp-BSA | 7.6 | 7.2 | 11.6 | 7.2 | 11.7 |  | 7.2 | 7.2 | 7.7 | 7.2 | 7.2 |
| 223 | Bdi-g - 06 | Gala1-3Galb– BSA | 13.2 | 12.9 | 11.8 | 8.2 | 12.5 |  | 9.8 | 9.1 | 7.9 | 7.2 | 7.2 |
| 224 | Lac-C5 - 14 | Galb1-4Glcb – BSA | 13.2 | 14.0 | 14.6 | 10.2 | 15.0 |  | 9.6 | 9.8 | 10.3 | 7.9 | 10.7 |
| 225 | Mana1-6Man-a - 04 | Mana1-6Man-a - BSA | 8.0 | 13.6 | 13.7 | 7.9 | 7.9 |  | 10.9 | 9.9 | 9.7 | 7.3 | 7.2 |
| 226 | B tetra type 1-Sp - 16 | Gala1-3[Fuca1-2]Galb1-3GlcNAcb-Sp-BSA | 12.6 | 13.5 | 9.9 | 12.4 | 13.1 |  | 9.3 | 9.7 | 7.2 | 9.1 | 7.7 |
| 227 | LacNAc-Sp - 06 | Galb1-4GlcNAcb-Sp-BSA | 8.7 | 7.2 | 12.7 | 7.2 | 9.3 |  | 7.2 | 7.2 | 8.5 | 7.2 | 7.2 |
| 228 | LNT-2-Sp - 15 | GlcNAcb1-3Galb1-4Glcb-Sp-BSA | 15.5 | 16.1 | 15.7 | 15.6 | 16.0 |  | 11.6 | 11.8 | 10.7 | 11.5 | 11.8 |
| 229 | GD1a-Sp - 05 | Neu5Aca2-3[Neu5Aca2-3Galb1-3GalNAcb1-4]Galb1-4Glcb-Sp-BSA | 8.5 | 10.2 | 10.4 | 12.5 | 13.8 |  | 7.2 | 7.7 | 7.2 | 9.1 | 9.3 |
| 230 | 6'Neu5Gc-LacNAc-Sp - 05 | Neu5Gca2-6Galb1-4GlcNAcb-Sp-BSA | 8.8 | 8.2 | 9.0 | 7.6 | 7.2 |  | 7.3 | 7.2 | 7.2 | 7.3 | 7.2 |
| 231 | SSEA-4-Sp - 05 | Neu5Aca2-3Galb1-3GalNAcb1-3Gala1-4Galb1-4Glcb-Sp-BSA | 11.5 | 9.1 | 12.8 | 8.3 | 12.4 |  | 9.4 | 7.2 | 9.4 | 7.3 | 7.7 |
| 232 | 3'Neu5Ac(9Ac)-LacNAc-Sp - 10 | Neu5Ac(9Ac)a2-3Galb1-4GlcNAcb-Sp-BSA | 11.2 | 11.8 | 13.4 | 9.2 | 13.8 |  | 8.6 | 8.8 | 9.5 | 7.6 | 9.2 |
| 233 | 3'Neu5Ac(9Ac)-LeC-Sp - 12 | Neu5Ac(9Ac)a2-3Galb1-3GlcNAcb-Sp-BSA | 13.5 | 12.4 | 12.5 | 10.6 | 13.0 |  | 9.8 | 9.1 | 8.6 | 8.2 | 8.0 |
| 234 | LDN-Sp - 14 | GalNAcb1-4GlcNAcb-Sp-BSA | 13.9 | 14.0 | 15.5 | 12.5 | 15.6 |  | 10.4 | 10.8 | 11.2 | 9.2 | 10.8 |
| 235 | B tetra type 2-Sp - 07 | Gala1-3[Fuca1-2]Galb1-4GlcNAcb-Sp-BSA | 9.8 | 9.9 | 10.1 | 7.9 | 7.8 |  | 7.8 | 7.8 | 7.2 | 7.5 | 7.2 |
| 236 | GD1a-Sp - 10 | Neu5Aca2-3[Neu5Aca2-3Galb1-3GalNAcb1-4]Galb1-4Glcb-Sp-BSA | 9.5 | 10.3 | 10.4 | 12.5 | 13.8 |  | 7.2 | 7.8 | 7.2 | 9.0 | 9.4 |
| 237 | Ac-S-Ser(GlcNAc-b)-S-G - 24 | Ac-Ser-(GlcNAcβ)Ser-Ser-Gly-Hex-BSA | 15.2 | 15.8 | 15.3 | 15.0 | 16.0 |  | 11.4 | 12.3 | 15.1 | 10.8 | 11.0 |
| 238 | LacNAc-Sp - 15 | Galb1-4GlcNAcb-Sp-BSA | 12.1 | 11.4 | 13.5 | 8.9 | 13.3 |  | 8.9 | 8.4 | 9.3 | 7.7 | 7.4 |
| 239 | LeC (dimeric)-Sp - 06 | Galb1-3GlcNAcb1-3Galb1-3GlcNAcb-Sp-BSA | 11.9 | 10.1 | 11.7 | 9.0 | 14.0 |  | 8.6 | 7.7 | 7.9 | 7.5 | 8.4 |
| 240 | Lac-C5 - 05 | Galb1-4Glcb – BSA | 14.2 | 14.8 | 16.1 | 12.5 | 16.1 |  | 10.1 | 10.1 | 11.2 | 9.5 | 10.6 |
| 241 | SSEA-4-Sp - 12 | Neu5Aca2-3Galb1-3GalNAcb1-3Gala1-4Galb1-4Glcb-Sp-BSA | 11.9 | 10.5 | 14.3 | 8.6 | 13.4 |  | 10.3 | 7.5 | 9.9 | 7.3 | 7.8 |
| 242 | Bdi-g - 16 | Gala1-3Galb– BSA | 13.1 | 13.2 | 13.4 | 10.5 | 13.3 |  | 10.5 | 10.0 | 9.4 | 8.2 | 9.7 |
| 243 | A tetra type 1-Sp - 15 | GalNAca1-3[Fuca1-2]Galb1-3GlcNAcb-Sp-BSA | 15.0 | 12.0 | 14.9 | 10.7 | 15.5 |  | 11.7 | 8.8 | 10.6 | 8.6 | 10.7 |
| 244 | Forssman Tetra-BSA - 05 | GalNAca1-3GalNAcb1-3Gala1-4Galb-BSA | 15.2 | 15.6 | 16.1 | 14.9 | 16.3 |  | 13.2 | 11.6 | 11.8 | 11.7 | 11.7 |
| 245 | BSA - C5 (Alkyne) - 10 | DF-168B-175-1 C5-alkyne-BSA | 10.7 | 10.5 | 11.0 | 9.1 | 11.6 |  | 8.6 | 7.9 | 8.3 | 7.8 | 7.6 |
| 246 | BG-H2 - 16 | Fuca1-2Galb1-4GlcNAcb-HSA | 10.1 | 10.9 | 12.5 | 9.2 | 11.6 |  | 9.4 | 9.3 | 8.7 | 7.6 | 8.0 |
| 247 | CT/Sda-Sp - 13 | Neu5Aca2-3[GalNAcb1-4]Galb1-4GlcNAcb-Sp-BSA; like GM2 but on glycoproteins | 10.4 | 11.0 | 11.7 | 8.7 | 12.8 |  | 9.0 | 8.4 | 8.7 | 7.3 | 8.1 |
| 248 | A tetra type 2-Sp - 17 | GalNAca1-3[Fuca1-2]Galb1-4GlcNAcb-Sp-BSA | 15.6 | 9.8 | 16.0 | 7.7 | 16.1 |  | 11.8 | 7.6 | 11.6 | 7.2 | 11.4 |
| 249 | 6'Neu5Ac-LacNAc (dimeric)-Sp - 13 | Neu5Aca2-6[Galb1-4GlcNAcb1-3)2b-Sp-BSA | 11.4 | 10.8 | 12.2 | 8.0 | 12.5 |  | 8.4 | 7.6 | 8.4 | 7.2 | 7.2 |
| 250 | GM3(Gc)-Sp - 14 | Neu5Gca2-3Galb1-4Glcb-Sp-BSA | 8.9 | 10.1 | 11.6 | 7.7 | 8.5 |  | 7.2 | 7.7 | 7.6 | 7.2 | 7.2 |
| 251 | 3'KDN-LacNAc-Sp - 05 | KDNa2-3Galb1-4GlcNAcb-Sp-BSA | 14.1 | 14.3 | 15.0 | 13.9 | 16.3 |  | 10.5 | 10.8 | 10.7 | 10.7 | 11.2 |
| 252 | GNLacNAc-Sp - 06 | GlcNAcb1-3Galb1-4GlcNAcb-Sp-BSA | 14.1 | 15.6 | 14.8 | 14.3 | 14.9 |  | 10.1 | 11.3 | 9.8 | 10.3 | 9.0 |
| 253 | 2'F-A type 2-Sp - 05 | GalNAca1-3[Fuca1-2]Galb1-4[Fuca1-3]GlcNAcb-Sp-BSA | 14.5 | 9.3 | 14.7 | 7.2 | 15.6 |  | 10.7 | 7.4 | 10.0 | 7.2 | 10.5 |
| 254 | Gb4 tetra (P1 tetra)-Sp - 15 | GalNAcb1-3Gala1-4Galb1-4GlcNAcb-Sp-BSA | 11.9 | 10.6 | 15.1 | 10.6 | 14.7 |  | 10.7 | 8.3 | 10.8 | 9.8 | 12.0 |
| 255 | GNLacNAc-Sp - 16 | GlcNAcb1-3Galb1-4GlcNAcb-Sp-BSA | 15.3 | 16.1 | 15.9 | 15.2 | 15.9 |  | 11.3 | 12.0 | 10.8 | 11.4 | 10.5 |
| 256 | 3'Neu5Gc-LacNAc-Sp - 06 | Neu5Gca2-3Galb1-4GlcNAcb-Sp-BSA | 8.6 | 7.4 | 11.7 | 8.2 | 8.8 |  | 7.5 | 7.2 | 7.8 | 7.2 | 7.2 |
| 257 | GT3-Sp - 03 | Neu5Aca2-8Neu5Aca2-8Neu5Aca2-3Galb1-4Glcb-Sp-BSA | 8.7 | 12.3 | 9.5 | 7.7 | 14.3 |  | 7.3 | 8.9 | 7.2 | 7.3 | 8.2 |
| 258 | Glc-a - 05 | Glc-a - BSA | 14.5 | 16.0 | 15.3 | 14.2 | 15.5 |  | 10.9 | 11.8 | 10.4 | 10.7 | 10.4 |
| 259 | LeC-Sp - 15 | Galb1-3GlcNAcb-Sp-BSA | 14.3 | 14.6 | 14.4 | 13.0 | 15.7 |  | 11.1 | 10.7 | 10.1 | 10.0 | 10.8 |
| 260 | Triazole linker from Xuefei - 43 | BSA-linker-triazole from Xuefei | 15.1 | 13.9 | 14.4 | 11.3 | 15.4 |  | 11.7 | 10.2 | 9.9 | 8.8 | 10.4 |
| 261 | 6'Neu5Ac-LacNAc-Sp - 05 | Neu5Aca2-6Galb1-4GlcNAcb-Sp-BSA | 10.6 | 10.2 | 10.8 | 8.9 | 12.2 |  | 8.6 | 8.0 | 8.0 | 7.7 | 7.5 |
| 262 | Fuc-GM1a - 08 | Fuca1-2Galb1-3GalNAcb1-4(Neu5Aca2-3)Galb1-4-BSA | 10.1 | 11.9 | 12.2 | 12.1 | 13.9 |  | 8.2 | 9.7 | 8.3 | 8.9 | 9.8 |
| 263 | 3'KDN-LeC-Sp - 04 | KDNa2-3Galb1-3GlcNAcb-Sp-BSA | 14.1 | 14.1 | 14.4 | 12.4 | 16.3 |  | 10.6 | 10.7 | 10.5 | 9.9 | 11.4 |
| 264 | Man1 - 04 | Manβ1-4GlcNAcβ1-4GlcNAcβ1-BSA | 13.0 | 15.1 | 15.1 | 10.2 | 15.5 |  | 10.0 | 10.2 | 10.3 | 8.0 | 10.2 |
| 265 | A tetra type 1-Sp - 05 | GalNAca1-3[Fuca1-2]Galb1-3GlcNAcb-Sp-BSA | 14.1 | 8.5 | 13.5 | 8.0 | 14.5 |  | 10.7 | 7.5 | 9.6 | 7.4 | 9.4 |
| 266 | 3'GN type1-Sp - 16 | GlcNAcb1-3Galb1-3GlcNAcb-Sp-BSA | 14.4 | 15.6 | 15.2 | 14.5 | 15.4 |  | 10.6 | 11.8 | 10.6 | 10.7 | 10.0 |
| 267 | 2'F-B type 2-Sp - 15 | Gala1-3[Fuca1-2]Galb1-4[Fuca1-3]GlcNAcb-Sp-BSA | 9.9 | 11.1 | 9.6 | 7.8 | 11.4 |  | 7.9 | 8.3 | 7.2 | 7.3 | 7.2 |
| 268 | 3'KDN-LeC-Sp - 12 | KDNa2-3Galb1-3GlcNAcb-Sp-BSA | 14.5 | 14.4 | 15.0 | 14.3 | 16.3 |  | 10.8 | 10.8 | 10.7 | 11.2 | 11.3 |
| 269 | B tetra type 2-Sp - 20 | Gala1-3[Fuca1-2]Galb1-4GlcNAcb-Sp-BSA | 11.2 | 11.9 | 11.9 | 8.6 | 11.5 |  | 8.4 | 8.7 | 7.8 | 7.5 | 7.2 |
| 270 | Galb1-4Gal | Galb1-4Gal-BSA | 15.5 | 15.4 | 15.2 | 14.5 | 16.2 |  | 11.2 | 10.8 | 10.5 | 10.5 | 10.7 |
| 271 | GD3-Sp - 04 | Neu5Aca2-8Neu5Aca2-3Galb1-4Glcb-Sp-BSA | 11.1 | 13.2 | 12.2 | 12.9 | 14.2 |  | 8.5 | 9.6 | 8.5 | 9.5 | 9.8 |
| 272 | 3'KDN-LacNAc-Sp - 12 | KDNa2-3Galb1-4GlcNAcb-Sp-BSA | 14.5 | 14.7 | 15.4 | 14.8 | 16.3 |  | 11.2 | 11.3 | 11.1 | 11.8 | 11.4 |
| 273 | 3'Neu5Ac-LacNAc-Sp - 05 | Neu5Aca2-3Galb1-4GlcNAcb-Sp-BSA | 10.2 | 9.2 | 12.5 | 7.2 | 12.9 |  | 8.0 | 7.3 | 8.7 | 7.2 | 7.5 |
| 274 | 6'Neu5Ac-LDN-Sp - 13 | Neu5Aca2-6GalNAcb1-4GlcNAcb-Sp-BSA | 12.1 | 14.4 | 13.8 | 11.8 | 14.3 |  | 9.2 | 11.1 | 9.7 | 9.2 | 9.2 |
| 275 | Man-a - 05 | Man-a - BSA | 10.2 | 11.6 | 14.3 | 7.4 | 11.0 |  | 8.1 | 8.2 | 9.9 | 7.2 | 7.2 |
| 276 | B tetra type 1-Sp - 04 | Gala1-3[Fuca1-2]Galb1-3GlcNAcb-Sp-BSA | 10.6 | 10.6 | 9.7 | 10.3 | 10.6 |  | 8.4 | 8.7 | 7.3 | 8.1 | 7.3 |
| 277 | A tetra type 2-Sp - 07 | GalNAca1-3[Fuca1-2]Galb1-4GlcNAcb-Sp-BSA | 14.1 | 8.4 | 15.0 | 7.3 | 15.2 |  | 10.7 | 7.3 | 10.8 | 7.2 | 10.6 |
| 278 | LNT-Sp - 06 | Galb1-3GlcNAcb1-3Galb1-4GlcNAcb-Sp-BSA | 10.6 | 10.8 | 11.9 | 8.5 | 12.5 |  | 8.2 | 8.2 | 8.4 | 7.4 | 7.9 |
| 279 | LeC-Sp - 07 | Galb1-3GlcNAcb-Sp-BSA | 12.3 | 12.3 | 12.5 | 10.1 | 13.9 |  | 9.4 | 9.2 | 9.0 | 8.3 | 9.1 |
| 280 | GQ2-Sp - 06 | Neu5Aca2-8Neu5Aca2-8Neu5Aca2-8Neu5Aca2-3[GalNAcb1-4]Galb1-4Glcb-Sp-BSA | 10.9 | 12.6 | 11.6 | 12.2 | 13.8 |  | 8.1 | 10.1 | 7.8 | 9.1 | 7.9 |
| 281 | 3'Neu5Ac-LeC-Sp - 12 | Neu5Aca2-3Galb1-3GlcNAcb-Sp-BSA | 11.7 | 11.5 | 12.2 | 10.3 | 13.2 |  | 8.7 | 8.5 | 8.3 | 8.1 | 7.8 |
| 282 | GM3-Sp - 11 | Neu5Aca2-3Galb1-4Glcb-Sp-BSA | 9.2 | 13.3 | 12.5 | 12.1 | 11.8 |  | 7.5 | 9.8 | 8.7 | 9.4 | 7.6 |
| 283 | R-Tn(Ser)-Tn-hydroxyethylamide - 36 | BSA-linker-Tn(Ser)-hydroxyethylamide | 16.4 | 15.6 | 16.4 | 15.3 | 16.4 |  | 12.7 | 11.8 | 12.0 | 11.9 | 11.7 |
| 284 | 3'Neu5Ac-LeC-Sp - 05 | Neu5Aca2-3Galb1-3GlcNAcb-Sp-BSA | 10.0 | 8.9 | 11.3 | 8.0 | 11.7 |  | 8.0 | 7.6 | 7.6 | 7.4 | 7.2 |
| 285 | GM3-Sp - 04 | Neu5Aca2-3Galb1-4Glcb-Sp-BSA | 8.8 | 12.3 | 11.6 | 11.6 | 11.3 |  | 7.7 | 9.3 | 8.0 | 9.1 | 7.3 |
| 286 | 3'GN-LacNAc (dimeric)-Sp - 14 | GlcNAcb1-3(Galb1-4GlcNAcb1-3)2b-Sp-BSA | 14.2 | 15.4 | 15.0 | 14.4 | 14.9 |  | 10.2 | 11.2 | 10.1 | 10.5 | 9.2 |
| 287 | LNT-2-Sp - 06 | GlcNAcb1-3Galb1-4Glcb-Sp-BSA | 14.4 | 15.6 | 14.7 | 14.5 | 15.5 |  | 10.7 | 11.5 | 9.8 | 10.6 | 10.9 |
| 288 | GT2-Sp - 08 | Neu5Aca2-8Neu5Aca2-8Neu5Aca2-3[GalNAcb1-4]Galb1-4Glcb-Sp-BSA | 9.4 | 12.3 | 11.8 | 11.5 | 12.9 |  | 8.1 | 10.0 | 8.0 | 8.5 | 7.6 |
| 289 | Forssman Tetra-BSA - 13 | GalNAca1-3GalNAcb1-3Gala1-4Galb-BSA | 15.9 | 16.2 | 16.4 | 15.5 | 16.4 |  | 14.1 | 12.2 | 12.5 | 12.6 | 12.4 |
| 290 | alpha-Gal tetra - 17 | Gala1-3Galb1-4GlcNAcb1-3Galb1-BSA | 15.1 | 15.7 | 15.4 | 12.3 | 16.2 |  | 11.9 | 11.9 | 11.5 | 9.7 | 12.3 |
| 291 | Gb5/SSEA3 - 04 | Galb1-3GalNAcb1-3Gala1-4Galb1-BSA | 8.9 | 10.3 | 10.0 | 7.8 | 13.3 |  | 7.4 | 7.2 | 7.4 | 7.2 | 8.5 |
| 292 | 3'Neu5Ac-LacNAc-Sp - 10 | Neu5Aca2-3Galb1-4GlcNAcb-Sp-BSA | 10.3 | 8.6 | 12.2 | 7.5 | 12.6 |  | 7.8 | 7.4 | 8.3 | 7.2 | 7.8 |
| 293 | Globo H - 10 | Fuca1-2Galb1-3GalNAcb1-3Gala1-4Galb1-BSA | 9.4 | 8.0 | 10.9 | 8.4 | 11.8 |  | 7.4 | 7.2 | 7.9 | 7.2 | 7.6 |
| 294 | Globo A - 09 | GalNAca1-3(Fuca1-2)Galb1-3GalNAcb1-3Gala1-4Galb1-BSA | 14.7 | 9.7 | 14.3 | 8.0 | 15.5 |  | 11.2 | 7.8 | 9.9 | 7.3 | 10.8 |
| 295 | GT2-Sp - 03 | Neu5Aca2-8Neu5Aca2-8Neu5Aca2-3[GalNAcb1-4]Galb1-4Glcb-Sp-BSA | 7.4 | 11.7 | 10.4 | 10.1 | 11.4 |  | 7.2 | 9.5 | 7.3 | 7.9 | 7.2 |
| 296 | 3'Neu5Gc-LacNAc-Sp - 10 | Neu5Gca2-3Galb1-4GlcNAcb-Sp-BSA | 7.2 | 7.5 | 12.4 | 7.2 | 9.0 |  | 7.2 | 7.2 | 8.2 | 7.2 | 7.2 |
| 297 | 3'Neu5Ac-LacNAc (dimeric)-Sp - 13 | Neu5Aca2-3(Galb1-4GlcNAcb1-3)2b-Sp-BSA | 13.4 | 8.0 | 11.8 | 7.2 | 8.7 |  | 9.3 | 7.2 | 8.0 | 7.2 | 7.2 |
| 298 | CT/Sda-Sp - 05 | Neu5Aca2-3[GalNAcb1-4]Galb1-4GlcNAcb-Sp-BSA; like GM2 but on glycoproteins | 9.2 | 9.0 | 10.9 | 7.4 | 12.3 |  | 7.9 | 7.5 | 7.8 | 7.2 | 7.8 |
| 299 | Ac-S-Ser(GlcNAc-a)-S-G - 07 | Ac-Ser-(GlcNAcα)Ser-Ser-Gly-Hex-BSA | 15.2 | 15.7 | 15.1 | 13.8 | 15.7 |  | 14.3 | 13.5 | 11.0 | 15.0 | 14.8 |
| 300 | TFiLNO(1-2,1-2,1-4) - 06 | Fuca1-2Galb1-3GlcNAcb1-3Galb1-4GlcNAcb1-6[Fuca1-2Galb1-3(Fuca1-4)GlcNAcb1-3]Galb-BSA | 13.6 | 11.2 | 11.5 | 7.5 | 12.1 |  | 9.8 | 8.4 | 7.7 | 7.3 | 7.2 |
| 301 | GD3-Sp - 08 | Neu5Aca2-8Neu5Aca2-3Galb1-4Glcb-Sp-BSA | 10.6 | 13.9 | 12.7 | 13.1 | 14.5 |  | 8.2 | 10.2 | 8.8 | 10.0 | 10.2 |
| 302 | LNT-Sp - 15 | Galb1-3GlcNAcb1-3Galb1-4GlcNAcb-Sp-BSA | 12.9 | 13.6 | 14.2 | 11.6 | 14.8 |  | 9.4 | 9.9 | 9.9 | 8.7 | 10.0 |
| 303 | 3'Neu5Gc-LeC-Sp - 12 | Neu5Gca2-3Galb1-3GlcNAcb-Sp-BSA | 11.7 | 10.1 | 11.1 | 9.6 | 12.3 |  | 9.0 | 7.7 | 7.7 | 7.9 | 7.7 |
| 304 | GQ2-Sp - 03 | Neu5Aca2-8Neu5Aca2-8Neu5Aca2-8Neu5Aca2-3[GalNAcb1-4]Galb1-4Glcb-Sp-BSA | 7.6 | 11.9 | 10.2 | 11.3 | 12.7 |  | 7.2 | 9.7 | 7.3 | 8.6 | 7.5 |
| 305 | Gb5/SSEA3 - 12 | Galb1-3GalNAcb1-3Gala1-4Galb1-BSA | 10.8 | 12.2 | 12.1 | 9.0 | 13.5 |  | 8.6 | 8.6 | 8.9 | 7.3 | 10.0 |
| 306 | Ac-S-Ser(GlcNAc-b)-S-G - 07 | Ac-Ser-(GlcNAcβ)Ser-Ser-Gly-Hex-BSA | 13.0 | 14.8 | 13.2 | 11.0 | 14.7 |  | 9.8 | 10.9 | 12.8 | 8.6 | 10.2 |
| 307 | LacNAc (dimeric)-Sp - 16 | (Galb1-4GlcNAcb1-3)2b-Sp-BSA | 10.1 | 11.1 | 12.4 | 7.8 | 13.0 |  | 7.5 | 7.9 | 8.5 | 7.2 | 7.7 |
| 308 | 3'Neu5Ac-LacNAc (dimeric)-Sp - 06 | Neu5Aca2-3(Galb1-4GlcNAcb1-3)2b-Sp-BSA | 10.4 | 7.2 | 10.7 | 7.2 | 7.2 |  | 7.2 | 7.2 | 7.5 | 7.2 | 7.2 |
| 309 | alpha-Gal tetra - 04 | Gala1-3Galb1-4GlcNAcb1-3Galb1-BSA | 12.0 | 11.3 | 11.4 | 7.3 | 15.1 |  | 9.5 | 8.3 | 8.1 | 7.2 | 11.4 |
| 310 | 3'Neu5Ac(9Ac)-LeC-Sp - 05 | Neu5Ac(9Ac)a2-3Galb1-3GlcNAcb-Sp-BSA | 11.0 | 10.9 | 11.1 | 7.4 | 8.8 |  | 8.2 | 8.2 | 7.3 | 7.2 | 7.2 |
| 311 | LacNAc (dimeric)-Sp - 06 | (Galb1-4GlcNAcb1-3)2b-Sp-BSA | 9.1 | 8.2 | 11.4 | 7.2 | 8.7 |  | 7.3 | 7.2 | 8.1 | 7.2 | 7.2 |
| 312 | GM2-Sp - 07 | Neu5Aca2-3[GalNAcb1-4]Galb1-4Glcb-Sp-BSA | 10.3 | 13.3 | 14.2 | 13.0 | 14.7 |  | 7.8 | 9.6 | 9.8 | 9.5 | 10.3 |
| 313 | MFiLNO(1-3) - 09 | Galb1-3GlcNAcb1-3Galb1-4(Fuca1-3)GlcNAcb1-6 (Galb1-3GlcNAcb1-3)Galb1-BSA | 11.7 | 12.8 | 14.0 | 10.3 | 13.9 |  | 9.2 | 9.8 | 9.4 | 7.9 | 8.9 |
| 314 | GD2-Sp - 04 | Neu5Aca2-8Neu5Aca2-3[GalNAcb1-4]Galb1-4Glcb-Sp-BSA | 7.8 | 11.9 | 12.5 | 7.4 | 10.9 |  | 7.6 | 9.9 | 8.6 | 7.2 | 7.4 |
| 315 | 6'Neu5Ac-LacNAc (dimeric)-Sp - 05 | Neu5Aca2-6[Galb1-4GlcNAcb1-3)2b-Sp-BSA | 10.0 | 10.1 | 11.2 | 7.2 | 9.5 |  | 7.8 | 7.8 | 7.9 | 7.2 | 7.2 |
| 316 | 3'Neu5Gc-LeC-Sp - 05 | Neu5Gca2-3Galb1-3GlcNAcb-Sp-BSA | 8.7 | 7.3 | 9.4 | 7.2 | 9.1 |  | 7.3 | 7.2 | 7.2 | 7.2 | 7.2 |
| 317 | DFLNnH, LeA/LeA - 10 | Galb1-4(Fuca1-3)GlcNAcb1-6[Galb1-4(Fuca1-3)GlcNAcb1-3]Galb1-BSA | 14.0 | 14.7 | 13.0 | 12.2 | 15.6 |  | 10.6 | 11.0 | 8.8 | 8.9 | 10.9 |
| 318 | GD1b - 05 | Neu5Aca2-8Siaa2-3(Galb1-3GalNAcb1-4)Galb1-4-BSA | 9.2 | 10.4 | 9.9 | 7.2 | 9.0 |  | 7.6 | 8.4 | 7.5 | 9.4 | 7.2 |
| 319 | 6'Neu5Ac-LacNAc-Sp - 11 | Neu5Aca2-6Galb1-4GlcNAcb-Sp-BSA | 9.4 | 11.1 | 11.6 | 8.4 | 13.0 |  | 7.5 | 7.9 | 10.8 | 7.2 | 7.8 |
| 320 | 3'GN-LacNAc (dimeric)-Sp - 06 | GlcNAcb1-3(Galb1-4GlcNAcb1-3)2b-Sp-BSA | 14.7 | 15.3 | 15.5 | 14.7 | 15.5 |  | 10.9 | 11.7 | 10.9 | 10.8 | 10.5 |
| 321 | Man1 - 12 | Manβ1-4GlcNAcβ1-4GlcNAcβ1-BSA | 14.5 | 15.7 | 16.0 | 12.9 | 16.2 |  | 10.7 | 11.0 | 11.2 | 9.4 | 10.9 |
| 322 | GM3(Gc)-Sp - 05 | Neu5Gca2-3Galb1-4Glcb-Sp-BSA | 7.9 | 9.6 | 9.6 | 7.2 | 9.1 |  | 7.2 | 7.2 | 7.2 | 7.2 | 7.2 |
| 323 | LDN-Sp - 05 | GalNAcb1-4GlcNAcb-Sp-BSA | 11.1 | 11.8 | 13.1 | 8.9 | 12.5 |  | 9.0 | 9.2 | 9.5 | 7.6 | 8.6 |
| 324 | 6'Neu5Ac-LDN-Sp - 05 | Neu5Aca2-6GalNAcb1-4GlcNAcb-Sp-BSA | 9.4 | 11.6 | 11.8 | 7.6 | 12.7 |  | 7.3 | 9.0 | 7.5 | 7.3 | 7.6 |
| 325 | GT3-Sp - 07 | Neu5Aca2-8Neu5Aca2-8Neu5Aca2-3Galb1-4Glcb-Sp-BSA | 10.9 | 11.5 | 10.4 | 9.0 | 13.5 |  | 8.4 | 8.6 | 7.4 | 7.5 | 8.8 |
| 326 | Ac-S-Thr(core 3)-S-G - 05 | Ac-Ser-(GlcNAcb1-3GalNAca)Thr-Ser-Gly-Hex-BSA | 13.0 | 15.7 | 14.8 | 13.9 | 14.5 |  | 9.4 | 11.9 | 10.4 | 10.1 | 8.8 |
| 327 | 3'Neu5Ac-LDN-Sp - 11 | Neu5Aca2-3GalNAcb1-4GlcNAcb-Sp-BSA | 11.8 | 13.4 | 14.8 | 10.3 | 14.5 |  | 9.1 | 10.0 | 10.5 | 8.1 | 9.9 |
| 328 | 3'GN type1-Sp - 04 | GlcNAcb1-3Galb1-3GlcNAcb-Sp-BSA | 12.7 | 15.1 | 14.4 | 13.4 | 14.0 |  | 9.8 | 11.5 | 9.9 | 9.9 | 8.8 |
| 329 | DFLNHc, LacNAc/LeB - 08 | Galb1-4GlcNAcb1-6[Fuca1-2Galb1-3(Fuca1-4)GlcNAcb1-3]Galb1-BSA | 8.2 | 7.7 | 10.2 | 7.8 | 8.3 |  | 7.5 | 7.3 | 7.2 | 7.2 | 7.2 |
| 330 | GD2-Sp - 10 | Neu5Aca2-8Neu5Aca2-3[GalNAcb1-4]Galb1-4Glcb-Sp-BSA | 10.5 | 12.4 | 13.2 | 8.7 | 12.2 |  | 8.8 | 10.3 | 9.0 | 7.8 | 7.9 |
| 331 | 3'Neu5Ac-LDN-Sp - 05 | Neu5Aca2-3GalNAcb1-4GlcNAcb-Sp-BSA | 11.4 | 12.9 | 14.5 | 9.6 | 14.3 |  | 9.1 | 9.9 | 10.2 | 7.8 | 10.1 |
| 332 | BSA - C5 (Alkyne) - 23 | DF-168C-16-B5 C5-alkyne-BSA | 8.3 | 10.1 | 11.1 | 7.4 | 12.2 |  | 8.0 | 8.4 | 8.1 | 7.5 | 8.4 |
| 333 | 2'F-B type 2-Sp - 03 | Gala1-3[Fuca1-2]Galb1-4[Fuca1-3]GlcNAcb-Sp-BSA | 7.6 | 8.6 | 8.8 | 7.2 | 7.5 |  | 7.3 | 7.6 | 7.2 | 7.2 | 7.2 |
| 334 | A tetra type 2-Sp - 05 | GalNAca1-3[Fuca1-2]Galb1-4GlcNAcb-Sp-BSA | 13.5 | 9.0 | 14.4 | 7.2 | 14.6 |  | 10.2 | 7.2 | 10.4 | 7.2 | 10.0 |
| 335 | B tetra type 2-Sp - 05 | Gala1-3[Fuca1-2]Galb1-4GlcNAcb-Sp-BSA | 8.4 | 7.9 | 8.9 | 7.2 | 8.0 |  | 7.2 | 7.2 | 7.2 | 7.2 | 7.2 |
| 336 | GM2-Sp - 04 | Neu5Aca2-3[GalNAcb1-4]Galb1-4Glcb-Sp-BSA | 8.0 | 12.3 | 10.9 | 12.4 | 13.7 |  | 7.4 | 8.9 | 8.1 | 9.2 | 9.9 |
| 337 | BG-A5-05 | GalNAca1-3(Fuca1-2)Galb1-3Galb1-linker-BSA | 14.0 | 8.3 | 11.1 | 8.4 | 12.1 |  | 10.7 | 7.2 | 7.9 | 7.4 | 7.9 |
| 338 | Ac-S-Thr(core 3)-S-G - 21 | Ac-Ser-(GlcNAcb1-3GalNAca)Thr-Ser-Gly-Hex-BSA | 15.4 | 16.3 | 15.9 | 15.7 | 16.1 |  | 11.5 | 12.5 | 11.6 | 11.8 | 11.1 |
| 339 | BG-B5-04 | Gala1-3(Fuca1-2)Galb1-3Galb1-linker-BSA | 10.5 | 14.3 | 11.0 |  | 12.0 |  | 7.2 | 9.6 | 7.7 |  | 7.2 |
| 340 | BG-B5-17 | Gala1-3(Fuca1-2)Galb1-3Galb1-linker-BSA | 13.8 | 12.4 | 13.4 | 11.2 | 14.4 |  | 10.5 | 9.1 | 9.2 | 9.5 | 8.0 |
| 341 | Ac-S-Thr(F1a)-S-G - 18 | AcSer-(Galb1-4GlcNAcb1-6GalNAca)Thr-Ser-Gly-Hex-BSA | 15.2 | 13.6 | 15.5 | 13.1 | 15.9 |  | 11.8 | 9.7 | 11.5 | 10.3 | 11.4 |
| 342 | BG-H1-10 | Fuca1-2Galb1-3GlcNAcb1-linker-BSA | 15.1 | 13.4 | 12.7 | 11.5 | 14.7 |  | 11.4 | 10.0 | 8.9 | 8.6 | 10.1 |
| 343 | BG-A6-04 | GalNAca1-3(Fuca1-2)Galb1-4Glcb1-linker-BSA | 11.3 | 9.4 | 10.6 | 7.9 | 11.9 |  | 8.4 | 7.4 | 7.6 | 7.2 | 7.4 |
| 344 | BG-A6-23 | GalNAca1-3(Fuca1-2)Galb1-4Glcb1-linker-BSA | 15.6 | 9.4 | 15.9 | 11.0 | 16.1 |  | 12.0 | 7.9 | 11.4 | 8.4 | 11.4 |
| 345 | BG-H5-04 | Fuca1-2Galb1-3Galb1-linker-BSA | 11.2 | 9.7 | 10.5 | 8.7 | 11.6 |  | 8.4 | 8.0 | 8.2 | 7.2 | 7.4 |
| 346 | BG-H5-19 | Fuca1-2Galb1-3Galb1-linker-BSA | 14.8 | 13.0 | 15.6 | 11.1 | 15.5 |  | 10.8 | 9.8 | 11.6 | 8.5 | 10.5 |
| 347 | BG-B6- 03 | Gala1-3(Fuca1-2)Galb1-4Glcb1-linker-BSA | 9.6 | 9.1 | 9.6 | 7.4 | 9.8 |  | 7.5 | 7.3 | 7.3 | 7.2 | 7.2 |
| 348 | BG-B6-15 | Gala1-3(Fuca1-2)Galb1-4Glcb1-linker-BSA | 11.0 | 12.4 | 13.0 | 10.0 | 13.6 |  | 8.7 | 9.1 | 9.7 | 8.2 | 8.9 |
| 349 | BG-H6-05 | Fuca1-2Galb1-4Glcb1-linker-BSA | 10.7 | 8.4 | 11.8 | 8.9 | 11.2 |  | 8.6 | 7.3 | 8.7 | 7.3 | 7.6 |
| 350 | BG-H6-21 | Fuca1-2Galb1-4Glcb1-linker-BSA | 12.8 | 12.1 | 15.5 | 12.5 | 14.2 |  | 11.7 | 10.6 | 11.0 | 9.3 | 9.6 |
| 351 | BG-A5-16 | GalNAca1-3(Fuca1-2)Galb1-3Galb1-linker-BSA | 16.0 | 11.3 | 16.0 | 9.7 | 16.2 |  | 12.3 | 8.1 | 11.6 | 7.4 | 11.6 |
| 352 | AGE60 | Advanced glycation endproducts day 60 | 7.5 | 7.2 | 7.7 | 7.2 | 8.6 |  | 7.2 | 7.2 | 7.2 | 7.2 | 7.2 |
| 353 | Alpha-1-acid glycoprotein | alpha1 Acid Glycoprotein-purified from human serum | 8.9 | 7.2 | 8.4 | 7.3 | 7.7 |  | 7.2 | 7.2 | 7.2 | 7.2 | 7.2 |
| 354 | Alpha-fetoprotein | alpha fetoprotein (AFP)-human, from cell culture | 8.6 | 9.1 | 10.6 | 8.8 | 8.8 |  | 7.3 | 7.4 | 7.8 | 8.8 | 7.2 |
| 355 | BSM | Bovine submaxillary mucin (STn, STF, S-GlcNAcb1-3, ~20% of Sia is acetylated at 7,8, or 9) | 11.8 | 13.6 | 13.9 | 9.9 | 13.7 |  | 9.3 | 10.3 | 10.4 | 8.2 | 9.9 |
| 356 | BSM (asialo) | Asialo-Bovine submaxillary mucin (aBSM, Tn, TF, GlcNAcb1-3GalNAc) | 11.0 | 12.4 | 12.1 | 12.3 | 11.1 |  | 8.5 | 9.6 | 8.4 | 9.7 | 9.1 |
| 357 | BSM (deacetylated) | Deacetylated-Bovine submaxillary mucin | 9.0 | 11.1 | 11.1 | 8.6 | 10.5 |  | 7.9 | 8.2 | 8.2 | 7.4 | 7.6 |
| 358 | BSM (ox) | periodate oxidized bovine submaxillary mucin | 10.0 | 10.7 | 10.5 | 8.8 | 11.2 |  | 8.5 | 8.5 | 8.0 | 7.5 | 8.2 |
| 359 | CEA | carcinoembryonic antigen isolated from human metastatic liver | 9.2 | 9.9 | 10.5 | 8.1 | 10.4 |  | 8.5 | 8.5 | 8.3 | 7.7 | 7.8 |
| 360 | FABP | Fatty Acid Binding Protein | 11.3 | 11.3 | 11.1 | 10.5 | 11.5 |  | 10.0 | 9.7 | 9.2 | 9.5 | 9.0 |
| 361 | fetuin | fetuin from calf serum (Neu5Ac2-3LacNAc, Neu5Ac2-6LacNAc, SiaLeC, STF) | 7.8 | 7.2 | 9.2 | 7.2 | 7.4 |  | 9.1 | 8.1 | 7.7 | 8.0 | 7.5 |
| 362 | fetuin (asialo) | asialofetuin from calf serum- type I (Galb1-4GlcNAc, Galb1-3GlcNAc, Galb1-3GalNAc; mostly NA2 and NA3) | 10.0 | 7.2 | 9.8 | 7.2 | 7.8 |  | 9.2 | 8.4 | 7.8 | 8.2 | 7.6 |
| 365 | Fetuin (ox) | periodate oxidized fetuin | 10.2 | 8.9 | 9.4 | 9.2 | 9.3 |  | 8.9 | 8.2 | 8.2 | 8.4 | 7.4 |
| 366 | glycophorin (asialo) | asialo-glycophorin A (aGn) | 10.6 | 11.5 | 11.2 | 10.3 | 12.7 |  | 8.1 | 8.4 | 8.1 | 8.2 | 8.4 |
| 367 | Glycophorin A | Glycophorin A (Gn) | 10.1 | 10.2 | 11.2 | 8.3 | 9.3 |  | 8.0 | 8.1 | 8.3 | 7.5 | 7.3 |
| 368 | hsp90 | Heat Shock Protein 90 | 7.2 | 14.9 | 9.3 | 7.2 | 8.9 |  | 8.2 | 11.7 | 8.6 | 8.8 | 8.0 |
| 369 | KLH | Keyhole limpet hemocyanin | 9.3 | 8.4 | 8.8 | 7.9 | 9.2 |  | 8.2 | 7.7 | 7.5 | 7.3 | 7.2 |
| 370 | gp120 | gp120 from HIV; expressed in HEK293T cells; LOTS OF CARRY OVER. WE NEED TO DOPE W BSA OR PUT A BLANK/BUFFER AFTER THIS (OR BOTH) | 7.2 | 7.9 | 8.0 | 8.6 | 7.2 |  | 7.9 | 8.0 | 8.3 | 9.1 | 7.6 |
| 371 | OSM | Ovine submaxillary mucin (94% STn, 4% TF, 2% Fuca1-2Galb1-3GalNAc) | 12.2 | 13.0 | 14.2 | 11.5 | 13.7 |  | 9.8 | 10.2 | 10.9 | 9.2 | 10.0 |
| 372 | OSM (asialo) | asialo-Ovine submaxillary mucin | 14.0 | 13.7 | 14.4 | 12.7 | 15.6 |  | 11.0 | 10.4 | 10.6 | 9.9 | 11.6 |
| 373 | OSM (ox) | periodate oxidized ovine submaxillary mucin | 12.9 | 12.3 | 13.1 | 11.6 | 14.1 |  | 10.1 | 9.4 | 10.1 | 8.8 | 10.4 |
| 374 | ovalbumin | ovalbumin (56% Man5+Man6) | 9.8 | 11.2 | 10.6 | 8.7 | 11.4 |  | 9.3 | 10.0 | 8.6 | 8.6 | 8.9 |
| 375 | Ovalbumin (ox) | periodate oxidized ovalbumin | 9.4 | 13.8 | 10.5 | 8.1 | 11.4 |  | 9.7 | 11.3 | 9.1 | 9.5 | 8.9 |
| 376 | PSA | Prostate Specific Antigen (PSA); human seminal fluid | 11.9 | 12.2 | 10.5 | 11.1 | 11.9 |  | 9.9 | 9.5 | 8.4 | 9.5 | 8.8 |
| 377 | Tgl | Thyroglobulin -human | 7.7 | 7.3 | 8.3 | 7.2 | 7.2 |  | 9.1 | 9.1 | 8.7 | 9.2 | 7.9 |
| 378 | FABP-3 | Fatty Acid Binding Protein (Heart type) | 10.4 | 10.6 | 10.6 | 9.3 | 10.5 |  | 8.7 | 8.6 | 8.7 | 8.0 | 7.8 |
| 379 | Ac-S-Thr(core 2)-S-G - 12 | Ac-Ser-(Galb1-3(GlcNAcb1-6)GalNAca)Thr-Ser-Gly-Hex-BSA | 14.0 | 15.4 | 14.6 | 14.1 | 15.0 |  | 10.0 | 11.2 | 10.2 | 10.9 | 10.0 |
| 380 | Ac-F-N(GlcNAcb1)-S-G-Hex-12 | AcPhe-(GlcNAcb1)Asn-Ser-Gly-Hex-12 | 14.9 | 15.3 | 14.9 | 12.2 | 15.7 |  | 11.0 | 11.3 | 10.2 | 9.4 | 11.2 |
| 381 | Ac-S-Ser(core 5)-S-G-04 | Ac-Ser-(GalNAca1-3GalNAca)Ser-Ser-Gly-Hex-BSA | 11.4 | 13.6 | 15.3 | 13.2 | 15.9 |  | 8.1 | 9.5 | 11.3 | 10.0 | 11.2 |
| 382 | Ac-S-Ser(core 5)-S-G-18 | Ac-Ser-(GalNAca1-3GalNAca)Ser-Ser-Gly-Hex-BSA | 15.5 | 15.9 | 16.4 | 15.3 | 16.4 |  | 11.8 | 11.8 | 12.3 | 11.9 | 12.1 |
| 383 | Ac-Ser(core 5)-S-G-05 | Ac-(GalNAca1-3GalNAca)Ser-Ser-Gly-Hex-BSA | 13.3 | 14.5 | 15.8 |  | 16.0 |  | 9.9 | 9.9 | 11.7 |  | 10.9 |
| 384 | Ac-Ser(core 5)-S-G-08 | Ac-(GalNAca1-3GalNAca)Ser-Ser-Gly-Hex-BSA | 15.1 | 15.7 | 16.3 | 15.0 | 16.4 |  | 11.7 | 11.4 | 12.2 | 11.6 | 11.7 |
| 385 | Man9 #2 | extra? | 9.8 | 7.9 | 9.8 | 7.3 | 9.7 |  | 7.7 | 7.3 | 7.5 | 7.2 | 7.2 |
| 386 | Ac-G-S(Fuca)-S-G-Hex-05 | Ac-G-S(Fuca)-S-G-Hex-BSA | 7.7 | 8.4 | 8.2 | 7.2 | 8.1 |  | 7.2 | 7.2 | 7.2 | 7.2 | 7.2 |
| 387 | Ac-G-S(Fuca)-S-G-Hex-15 | Ac-G-S(Fuca)-S-G-Hex-BSA | 12.5 | 15.0 | 14.4 | 11.4 | 13.5 |  | 9.3 | 11.8 | 9.8 | 8.6 | 8.7 |
| 388 | Ac-G-S(Xylb)-G-G-Hex-04 | Ac-Gly-Ser(Xylb)-Gly-Gly-Hex-BSA | 14.0 | 15.5 | 14.5 | 12.5 | 14.9 |  | 10.6 | 11.2 | 9.9 | 9.2 | 9.7 |
| 389 | Ac-G-S(Xylb)-G-G-Hex-21 | Ac-Gly-Ser(Xylb)-Gly-Gly-Hex-BSA | 15.7 | 16.4 | 16.4 | 15.8 | 16.2 |  | 12.1 | 12.2 | 11.9 | 11.8 | 11.4 |
| 390 | Ac-G-S-T(Tna)A-P-G-Hex-06 | Ac-G-S-T(GalNAca)A-P-G-Hex-BSA | 11.7 | 13.0 | 14.9 | 9.6 | 13.4 |  | 9.3 | 9.7 | 10.4 | 7.9 | 8.7 |
| 391 | Ac-G-S-T(Tna)A-P-G-Hex-19 | Ac-G-S-T(GalNAca)A-P-G-Hex-BSA | 14.5 | 14.7 | 16.4 | 12.3 | 15.8 |  | 11.2 | 11.3 | 12.0 | 9.6 | 11.1 |
| 392 | Ac-A-S(Glcb)-S-G-Hex-05 | Ac-A-S(Glcb)-S-G-Hex-BSA | 12.9 | 15.7 | 14.3 | 11.7 | 13.7 |  | 10.3 | 11.8 | 9.3 | 9.1 | 8.2 |
| 393 | Ac-A-S(Glcb)-S-G-Hex-18 | Ac-A-S(Glcb)-S-G-Hex-BSA | 15.5 | 16.5 | 16.3 | 16.1 | 16.1 |  | 12.4 | 12.4 | 11.6 | 12.1 | 11.0 |
| 394 | Man8D1D3 #2 | Mana1-2Mana1-6(Mana1-3)Mana1-6(Mana1-2Mana1-2Mana1-3)Manb1-4GlcNAc-BSA | 10.2 | 11.3 | 11.2 | 7.3 | 10.6 |  | 8.3 | 8.9 | 8.0 | 7.2 | 7.2 |
| 395 | Muc1 | BSA--hexyl-G-V-T-S-A-P-D-T-R-P-A-P-G-S-T-A-P-P-A-amide | 11.2 | 12.0 | 10.2 | 7.2 | 12.2 |  | 8.1 | 8.6 | 7.6 | 7.3 | 7.7 |
| 396 | Muc1-Tn8 | BSA--hexyl-G-V-T-S-A-P-D-T(GalNAc-a)-R-P-A-P-G-S-T-A-P-P-A-amide | 9.9 | 13.2 | 9.8 | 7.4 | 12.0 |  | 8.1 | 11.0 | 7.5 | 7.5 | 8.0 |
| 397 | Muc1-Tn15 | BSA--hexyl-G-V-T-S-A-P-D-T-R-P-A-P-G-S-T(GalNAc-a)-A-P-P-A-amide | 11.1 | 14.0 | 12.1 | 9.1 | 12.5 |  | 8.3 | 10.2 | 8.4 | 7.7 | 7.8 |
| 398 | human IgM | use 50ug/mL + 75ug/mL BSA;from Saddam's data, it appears to have a little IgG and IgA in it. | 14.4 | 14.5 | 14.5 | 14.6 | 14.3 |  | 9.1 | 8.8 | 8.9 | 9.4 | 8.1 |
| 399 | human IgA | use 50ug/mL + 75ug/mL BSA | 13.5 | 13.7 | 13.0 | 12.8 | 13.4 |  | 8.9 | 8.9 | 9.0 | 9.1 | 8.0 |
| 400 | human IgG | use 25ug/mL + 100ug/mL BSA | 11.5 | 10.8 | 11.1 | 10.9 | 10.7 |  | 13.9 | 13.7 | 13.1 | 13.8 | 13.1 |
| 401 | Alexa Fluoro 647 | Alexa Fluoro 647-BSA (30mg/mL+ BSA, 125mg/mL total) | 12.5 | 12.7 | 12.8 | 12.1 | 14.8 |  | 12.1 | 12.2 | 11.7 | 11.6 | 12.1 |
| 402 | rabbit IgG | use 25ug/mL + 100ug/mL BSA; | 11.0 | 10.6 | 10.5 | 9.4 | 10.7 |  | 11.4 | 11.6 | 11.7 | 11.8 | 11.4 |
| 403 | mouse IgM | use 50ug/mL + 75ug/mL BSA; monoclonal IgM purified from hybridoma | 13.5 | 13.4 | 13.5 | 13.0 | 13.8 |  | 10.0 | 10.0 | 10.8 | 10.0 | 10.0 |
| 404 | mouse IgG | use 25ug/mL + 100ug/mL BSA | 11.6 | 11.0 | 11.6 | 10.2 | 11.3 |  | 10.0 | 9.9 | 9.6 | 9.8 | 8.7 |
| 405 | BSA #2 | Bovine serum albumin | 9.4 | 9.0 | 9.8 | 8.1 | 9.3 |  | 7.9 | 8.0 | 8.1 | 8.1 | 7.3 |
| 406 | Ac-S-S(Mana)-S-G - 06 | Ac-S-S(Man a)-S-G-Hex-OH | 13.7 | 10.9 | 11.0 | 9.4 | 11.0 |  | 10.1 | 8.4 | 9.0 | 8.8 | 7.9 |
| 407 | Ac-S-S(Gala)-S-G - 06 | Ac-S-S(Gal a)-S-G-Hex-OH | 12.0 | 13.6 | 11.9 | 9.5 | 11.1 |  | 10.4 | 10.1 | 8.8 | 8.6 | 7.9 |
| 408 | Ac-P-T(Mana)-A-G - 07 | Ac-Pro-Thr(Man a)-Ala-Gly-Hex-BSA | 8.9 | 8.1 | 9.0 | 7.2 | 9.6 |  | 7.6 | 7.5 | 7.8 | 7.9 | 7.5 |
| 409 | Ac-S-S(Mana)-S-G - 20 | Ac-S-S(Man a)-S-G-Hex-OH | 14.2 | 13.1 | 14.8 | 9.7 | 13.8 |  | 9.9 | 9.6 | 10.2 | 8.4 | 8.4 |
| 410 | Ac-S-S(Gala)-S-G - 16 | Ac-S-S(Gal a)-S-G-Hex-OH | 14.1 | 14.8 | 14.4 | 12.4 | 15.0 |  | 13.1 | 10.9 | 10.1 | 10.0 | 11.3 |
| 411 | Ac-P-T(Mana)-A-G - 22 | Ac-Pro-Thr(Man a)-Ala-Gly-Hex-BSA | 12.0 | 13.5 | 11.6 | 8.2 | 14.5 |  | 8.8 | 9.2 | 8.4 | 7.9 | 8.5 |
